# Exploration of Multidimensional Structural Optimization and Regulation Mechanisms: Catalysts and Reaction Environments in Electrochemical Ammonia Synthesis

**DOI:** 10.1002/advs.202416053

**Published:** 2025-01-31

**Authors:** Kaibin Chu, Bo Weng, Zhaorui Lu, Yang Ding, Wei Zhang, Rui Tan, Yu‐Ming Zheng, Ning Han

**Affiliations:** ^1^ School of Materials Science and Engineering Linyi University Linyi 276000 P. R. China; ^2^ State Key Laboratory of Advanced Environmental Technology Institute of Urban Environment, Chinese Academy of Sciences 1799 Jimei Road Xiamen 361021 P. R. China; ^3^ College of Resources and Environment University of Chinese Academy of Sciences 19 A Yuquan Road Beijing 100049 P. R. China; ^4^ College of Materials and Environmental Engineering Hangzhou Dianzi University Hangzhou Zhejiang 310018 China; ^5^ Institute of Energy Materials Science University of Shanghai for Science and Technology Shanghai 200093 P. R. China; ^6^ Department of Chemical Engineering Swansea University Swansea SA1 8EN UK; ^7^ The Edward S. Rogers Department of Electrical and Computer Engineering University of Toronto Toronto ON M5S 3G4 Canada

**Keywords:** electrocatalysts, electrochemical ammonia synthesis, material design strategies, reaction mechanisms

## Abstract

Ammonia (NH_3_) is esteemed for its attributes as a carbon‐neutral fuel and hydrogen storage material, due to its high energy density, abundant hydrogen content, and notably higher liquefaction temperature in comparison to hydrogen gas. The primary method for the synthetic generation of NH_3_ is the Haber–Bosch process, involving rigorous conditions and resulting in significant global energy consumption and carbon dioxide emissions. To tackle energy and environmental challenges, the exploration of innovative green and sustainable technologies for NH_3_ synthesis is imperative. Rapid advances in electrochemical technology have created fresh prospects for researchers in the realm of environmentally friendly NH_3_ synthesis. Nevertheless, the intricate intermediate products and sluggish kinetics in the reactions impede the progress of green electrochemical NH_3_ synthesis (EAS) technologies. To improve the activity and selectivity of the EAS, which encompasses the electrocatalytic reduction of nitrogen gas, nitrate, and nitric oxide, numerous electrocatalysts and design strategies have been meticulously investigated. Here, this review primarily delves into recent progress and obstacles in EAS pathways, examining methods to boost the yield rate and current efficiency of NH_3_ synthesis via multidimensional structural optimization, while also exploring the challenges and outlook for EAS.

## Graphical abstract

Ammonia (NH_3_) is favored for its carbon‐neutral fuel and hydrogen storage capabilities due to its high energy density and abundant hydrogen content. The traditional Haber–Bosch process for NH_3_ synthesis is energy‐intensive and emits carbon dioxide. Innovations in green electrochemical technologies offer a promising solution, although challenges remain in enhancing efficiency and overcoming reaction obstacles.

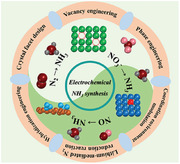

## 1 Introduction

**Figure 1:**
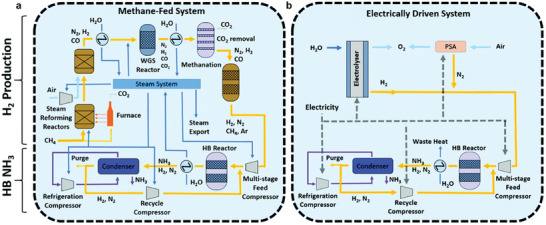
a) A typical H–B process fed by methane and b) an electrically‐driven alternative. Process gas, water/steam, air, NH_3_, and electricity are illustrated by yellow, dark blue, light blue, purple, and dashed lines, respectively. Reproduced with permission.^[^ ^4^ ^]^ Copyright 2020, Royal Society of Chemistry.

Ammonia (NH_3_) is a crucial chemical used in the fields of agriculture, healthcare, and energy.^[^ ^1^ ^]^ Specifically, NH_3_ is highly valued as a carbon‐neutral fuel and hydrogen storage material due to its high energy density (4.32 kWh L^−1^), rich hydrogen content (17.6 wt%), and significantly higher liquefaction temperature (−33 °C) compared to hydrogen gas (−253 °C).^[^ ^2^, ^3^ ^]^ Currently, the main method for artificial production of NH_3_ is the Haber–Bosch (H–B) process (**Figure** **1**a),^[^ ^4^ ^]^ which requires harsh conditions (e.g., 350–450 °C and 100–200 bar) to break the highly inert N≡N bond. Furthermore, this process accounts for 1% of global annual energy consumption and generates more than 1.4% of global carbon dioxide (CO_2_) emissions.^[^ ^5^, ^6^, ^7^ ^]^ This is mainly due to the hydrogen gas needed for NH_3_ synthesis being derived from the steam reforming of natural gas, resulting in the release of ≈2.86 metric tons of CO_2_ for every metric ton of NH_3_ produced.^[^ ^8^ ^]^

To address the challenges in energy and the environment effectively, it is necessary to explore novel green and sustainable technologies for NH_3_ synthesis that can mitigate the problems of high energy consumption and emissions. For example, the green hydrogen gas produced by water splitting has been utilized in the H–B process to avoid high emissions caused by natural gas reforming (Figure 1b).^[^ ^4^, ^9^ ^]^ However, it is faced with various challenges, including the improvement of the energy efficiency of water electrolysis, the investigation of advanced H–B processes under lower pressure conditions, and the exploration of alternative techniques for NH_3_ separation. The rapid advancement of electrochemical technology has opened up new opportunities for researchers in the field of green NH_3_ synthesis, which offers several advantages compared to traditional industrial approaches.^[^ ^10^, ^11^, ^12^, ^13^, ^14^, ^15^, ^16^, ^17^, ^18^ ^]^ First, it employs electrical energy as the driving force instead of thermal energy, facilitating reactions under milder conditions (lower temperature and pressure than the H–B process).^[^ ^11^ ^]^ Second, it requires less factory space and can utilize electricity generated from solar or wind sources, in contrast to the extensive footprint and complex process of the H–B process.^[^ ^19^ ^]^ Finally, using water as a proton source can effectively avoid location constraints caused by natural gas availability. Although electrochemical NH_3_ synthesis (EAS) has these advantages, the complex intermediate products and slow kinetics involved in the reactions lead to low yield rates and selectivity, which has been the major bottleneck hindering the development of green EAS technologies.^[^ ^20^, ^21^, ^22^, ^23^, ^24^, ^25^, ^26^, ^27^, ^28^ ^]^ Take electrochemical nitrogen gas (N_2_) reduction reaction (eN_2_RR) as an example, N_2_ from air was recognized as one major nitrogen source for green EAS. However, eN_2_RR encounters difficulties in achieving high activity due to the extremely stable N≡N triple bond (941 kJ mol^−1^) and low solubility of N_2_ in the electrolyte.^[^ ^29^, ^30^ ^]^ Similarly, EAS using other nitrogen‐containing compounds (NO, NO_2_^−^, and NO_3_^−^) as nitrogen sources also faces numerous challenges (such as low NH_3_ yield rates and poor selectivity, etc.).^[^ ^31^, ^32^, ^33^, ^34^, ^35^ ^]^

To enhance the activity and selectivity, various electrocatalyst, and design strategies have been explored for EAS, encompassing eN_2_RR, electrochemical nitric oxide (NO) reduction reaction (eNORR), and electrochemical nitrate (NO_3_^−^) reduction reaction (eNO_3_RR). Building on these advancements, researchers are increasingly focusing their efforts on discovering more efficient electrocatalysts and optimizing reaction environments, including reactors, electrolytes, and electrode designs, to enhance electrochemical NH_3_ synthesis. Hence, it is imperative to elucidate the mechanism and catalyst design strategies for EAS. Based on the aforementioned considerations, this review primarily focuses on recent advancements and challenges in the three pathways of EAS: 1) eN_2_RR, 2) eNORR, and 3) eNO_3_RR. Several strategies via multidimensional structural optimization, such as electrocatalyst design and electrochemical reactor engineering, are discussed to enhance the yield rate and current efficiency of NH_3_ synthesis. Additionally, the challenges and perspectives for EAS are also discussed.

## 2 The Pathway of EAS

### 2.1 Electrochemical Pathways of N_2_ Reduction

In general, the eN_2_RR in aqueous solution may follow two distinct reaction paths: one is the six‐electron transfer process that converts one N_2_ molecule into two NH_3_ molecules, and the other is the four‐electron transfer process that forms one hydrazine (N_2_H_4_) molecule.^[^ ^10^, ^36^ ^]^ The eN_2_RR to produce NH_3_ consists of three reaction processes: 1) adsorption of N_2_ molecules on the catalyst surface; 2) cleavage of the N≡N bond and hydrogenation of nitrogen atoms; 3) desorption of formed NH_3_ molecules or other intermediates from the catalyst surface. In the electrochemical process, the hydrogenation of N_2_ molecules is a continuous process involving both proton and electron transfers, where the electrolyte provides proton sources and the cathodic current supplies electrons.

**Figure 2:**
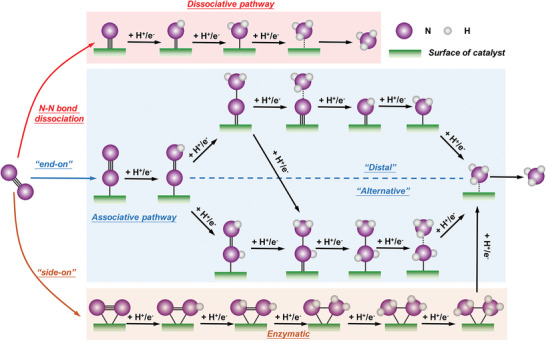
The reaction pathways for the eN_2_RR by proton‐coupled electron transfer include the dissociative pathway, distal/alternative associative pathway, and enzymatic pathway.

Based on the sequence of N≡N bond cleavage, two types of reaction mechanisms can be summarized: dissociative pathway and associative pathway,^[^ ^36^ ^]^ as illustrated in **Figure** **2**. Experimental evidence indicates that the H–B method for NH_3_ synthesis is based on a dissociative mechanism.^[^ ^37^ ^]^ The cleavage of the N≡N bond occurs after adsorption on the catalytic surface, preceding the hydrogenation step. For the associative alternating pathway, N≡N bond cleavage and hydrogenation processes occur simultaneously. It is assumed that the adsorption pattern of the N_2_ molecule is such that one end (one nitrogen atom) is adsorbed on the catalytic surface, allowing the hydrogenation process to be divided into two pathways: associative alternating pathway and associative distal pathway. In the associative alternating pathway, the hydrogenation process alternates between the two nitrogen atoms until the nitrogen–nitrogen bond breaks at the end, ultimately releasing two NH_3_ molecules. In the associative distal pathway, the uncoordinated nitrogen atom on the catalyst surface undergoes hydrogenation first until complete hydrogenation, and after the N≡N bond cleavage, one NH_3_ molecule is released, followed by the subsequent catalytic hydrogenation process of the remaining adsorbed nitrogen atom on the surface. In comparison to NH_3_, the production of N_2_H_4_ is proven to occur in the alternating pathway or in biological nitrogen fixation processes. However, the desorption process of *N_2_H_4_ is an endothermic reaction that requires a higher energy, making the generation of N_2_H_4_ less favorable.^[^ ^38^ ^]^

**Figure 3:**
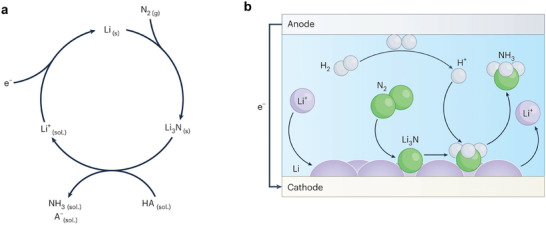
a) A schematic representation of the Li‐eN_2_RR cycle, with “sol.” denoting a solution, “s” representing a solid, and “g” indicating a gas. b) A schematic depiction of the cathode and anode reactions. Reproduced with permission.^[^ ^39^ ^]^ Copyright 2024, Springer Nature.

In recent years, numerous research studies on the eN_2_RR in aqueous solution have been published. However, the low solubility of N_2_, the chemical inertness of the N≡N bond, and competing HER in electrolyte solutions have posed challenges in achieving breakthroughs in both the yield rate and Faradaic efficiency (FE) of NH_3_ synthesis. Consequently, alternative approaches for cleaving the N≡N bond have been investigated. Leveraging the extremely low work function and high reactivity of lithium (Li), the use of Li as a mediator for N_2_ reduction can facilitate the reaction between Li and inert N_2_, resulting in the generation of Li_3_N. This, in turn, reduces the energy required for N_2_ cleavage, fundamentally enhancing the yield rate and FE of EAS at ambient temperature and pressure. Consequently, besides the conventional electrochemical conversion of N_2_ to NH_3_ in aqueous systems, a novel method known as Li‐mediated electrochemical N_2_ reduction reaction (Li‐eN_2_RR) has emerged. This approach is distinctive because both the N_2_ reduction and protonation processes occur within and depend on the solid electrolyte interphase (SEI) layer, which plays a crucial role in mediating catalysis. In this system, the electrochemical deposition of metallic Li in the presence of N_2_ leads to the formation of Li_3_N, which subsequently reacts with available protons to produce NH_3_ (**Figure** **3**).^[^ ^39^ ^]^

### 2.2 Electrochemical Pathways of NO_3_^−^ Reduction

eNO_3_RR is a complex multielectron transfer process involving various nitrogen‐containing intermediates (with valence of nitrogen ranging from +5 to −3). N_2_ and NH_3_ are the thermodynamically stable products of this process.^[^ ^40^, ^41^ ^]^ From an environmental perspective, N_2_, which is harmless to the environment, is the optimal product. From a “waste‐to‐value” perspective, NH_3_ is the optimal product. The equations for the reactions producing these two products are as follows^[^ ^10^ ^]^

(1) $$2{{\mathrm{NO}}_{3}}^{-}+12H^{+}+10e^{-}\to N_{2}+6H_{2}OE^{o}=1.17\;\mathrm{vs}\;\mathrm{SHE}$$

(2) $${{\mathrm{NO}}_{3}}^{-}+9H^{+}+8e^{-}\to {\mathrm{NH}}_{3}+3H_{2}O\;E^{o}=-0.12\;\mathrm{vs}\;\mathrm{SHE}$$

eNO_3_RR can be divided into two main parts: indirect spontaneous catalytic reduction pathway and direct catalytic reduction pathway. When NO_3_^−^ does not participate in the electron transfer process, the reaction proceeds through the indirect spontaneous catalytic reduction pathway.^[^ ^42^, ^43^ ^]^ The direct electrochemical reduction of NO_3_^−^ includes two pathways: one is the adsorption of active hydrogen for reduction, and the other is the electron reduction on the cathode.

**Figure 4:**
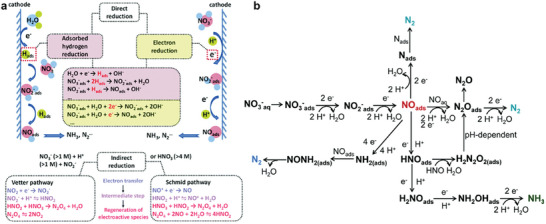
a) Illustration of the proposed direct and indirect pathways for the eNO_3_RR. b) Schematic of the electron‐mediated pathway for the eNO_3_RR. Reproduced with permission.ref. [10] Copyright 2021, Royal Society of Chemistry.

For the reduction of NO_3_^−^ through the adsorption of the hydrogen pathway, electrons first reduce the water molecules adsorbed on the cathode surface to form adsorbed hydrogen on the cathode surface, which then directly reduces NO_3_^−^ to NH_3_. As shown in **Figure** **4**a, various intermediates are generated after continuous reduction by active the hydrogen, such as NO_2_^−^_ads_, NO_ads_, N_ads_, NH_ads_, NH_2ads_, etc.^[^ ^44^ ^]^ It is evident that N_ad_ intermediates are formed in this process, but the energy barrier for N_ad_ migration is 0.75 eV, which is much higher than the energy barrier for H_ad_ (0.10 eV). Moreover, from a kinetic perspective, N*─*H bonds are easier to form compared to N*─*N bonds.^[^ ^45^ ^]^ As a result, the enhanced adsorption of H_ad_ on the catalyst surface favors the production of NH_3_.

The electron reduction mechanism is shown in Figure 4b. In the first step, NO_3_^−^ is converted to NO_2_^−^_ads_,^[^ ^46^, ^47^, ^48^, ^49^ ^]^ and in the second step, NO_2_^−^_ads_ are converted to NO_ads_. NO_ads_ is an important intermediate, and through this intermediate, N_2_ or NH_3_ can be further produced. There are multiple pathways for N_2_ formation. The first pathway involves the direct reduction of NO_ads_ to dissociatively adsorbed N_ads_, which combine to form N_2_.^[^ ^50^ ^]^ Another pathway involves the reaction between NO_ads_ and NO_aq_ in the solution to produce an N_2_O_ads_ intermediate,^[^ ^51^, ^52^ ^]^ which, once reduced on the catalyst surface, can also produce N_2_.^[^ ^53^, ^54^ ^]^ In addition, when NO_ads_ is further reduced by electrons, HNO is formed.^[^ ^55^ ^]^ Under suitable acidic conditions, HNO dimerizes to form a stable H_2_N_2_O_2_ intermediate.^[^ ^56^ ^]^ However, HN_2_O_2_^−^ can be converted to N_2_O in suitable pH ranges and then reduced to N_2_. Furthermore, under appropriate potential ranges, NO_ads_ can be directly reduced to NH_2ads_,^[^ ^57^, ^58^ ^]^ which can combine with NO_ads_ to generate a NONH_2_ intermediate, leading to N_2_ formation. Of course, the NO_ads_ intermediate can also continue to produce NH_3_ through another pathway, which is the desired process. Specifically, NO_ads_ is reduced by electrons to produce HNO_ads_, which then further produce H_2_NO_ads_ and H_2_NOH_ads_ intermediates through the further action of electron reduction, eventually producing NH_3_.

### 2.3 Electrochemical Pathways of NO Reduction

**Figure 5:**
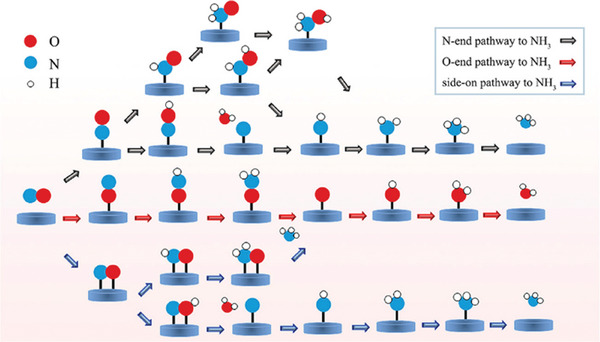
Schematic pathways of the eNORR process toward NH_3_ synthesis. Reproduced with permission.^[^ ^59^ ^]^ Copyright 2022, American Chemical Society.

As shown in **Figure** **5**, for eNORR, the way that NO reduces to NH_3_ in electrocatalysis may differ based on where the NO molecules attach to the surface of the catalyst. NO attachment locations include the end of the N (N‐end), end of the O (O‐end), and side‐on.^[^ ^59^ ^]^ The possible reaction pathways fall under two categories: dissociative and associative mechanisms. The dissociative mechanism breaks the N*─*O bond at the active site which leads to separate hydrogenation of the resulting N* and O* molecules. The associative mechanism involves the initial hydrogenation of NO molecules to form H*_x_* NOH*_y_* intermediates that are then stepwise hydrogenated to result in the release of NH_3_ and H_2_O. The hydrogenation of adsorbed atoms in the electrochemical NO‐to‐NH_3_ process happens through the Tafel or Heyrovsky mechanism. In the Tafel‐type route, solvated protons absorb onto the catalyst surface forming adsorbed H*. This is then followed by surface hydrogenation. In contrast, the adsorbed NO molecules and intermediate species are hydrogenated directly right from the start in the Heyrovsky‐type route. As a result, the eNORR mechanisms are divided into four categories depending on the type of hydrogenation: Tafel‐dissociative (T‐D), Tafel‐associative (T‐A), Heyrovsky‐dissociative (H‐D), and Heyrovsky‐associative (H‐A) mechanisms. T‐A and H‐A have four specific routes allocated depending on the adsorption type associated with each pathway; these are named: distal‐O, distal‐N, alternating‐O, and alternating‐N.^[^ ^60^ ^]^

## 3 Theoretical Insight for Electrocatalyst Design

In the realm of intricate electrochemical reactions, theoretical insights play a pivotal role in both elucidating reaction mechanisms and facilitating the design of high‐efficiency electrocatalysts.^[^ ^61^ ^]^ In‐depth theoretical computations have facilitated the emergence of multiple theories for optimizing the design of efficient electrocatalysts.^[^ ^62^, ^63^ ^]^ In this section, we provide a comprehensive overview of the theories and their applications in the development of EAS catalysts. Notably, Gibbs free energy change (Δ*G*) serves as a critical factor in characterizing the thermodynamic behavior of EAS. It has been suggested that the adsorption energy of reaction intermediates (such as *N_2_, *NNH, *NHNH, etc.) plays a substantial role in determining the electrocatalytic reaction dynamics on the surface of electrocatalysts.^[^ ^64^, ^65^ ^]^ Based on the Sabatier principle, ideal electrocatalysts should demonstrate suitable adsorption energy for both reactants and intermediates. Moreover, the *d*‐band theory offers a quantitative explanation of the adsorption interactions between catalysts and intermediates from an electronic standpoint, allowing for the modulation of active sites to improve inherent electrocatalytic performance. According to crystal field theory, the spin state further refines the d orbitals of electrocatalysts, which is directly linked to the adsorption strength of intermediates on the electrocatalyst surface. Therefore, identifying and adjusting active centers can enhance the electrochemical performance in electrocatalytic reactions.

Developed by Norskov et al.,^[^ ^66^ ^]^ the computational hydrogen electrode model enables the determination of Δ*G* for each step in the EAS process. Specifically, Δ*G* is calculated using the following equations

(3) $$\Delta G=\Delta E+\Delta ZPE-T\Delta S+\Delta S_{U}$$

(4) $$\Delta E=E_{T}-E_{\mathrm{catalyst}}-E_{\mathrm{adsorbate}}$$

Here, Δ*E* denotes the adsorption energy of the reactant N_2_ and corresponding intermediates, which is calculated using density functional theory (DFT). ΔZPE represents the change in zero‐point energy, *T*Δ*S* indicates the change in entropy at the specified temperature, and Δ*G*_U_ reflects the influence of the electrode potential (U) on Δ*G*. *E*_T_ is the total energy during EAS, *E*_catalyst_ is the free energy of the electrocatalyst, and *E*_adsorbate_ is the free energy of the adsorbed reactant. From a thermodynamic perspective, the elementary reaction with the maximum free energy change (Δ*G*_max_) is recognized as the rate‐limiting step. By analyzing Δ*G*_max_ values, the reaction pathways and EAS activity can be assessed. The Δ*G* values of intermediates can help identify the most favorable reaction pathway and the most active catalyst.

This highlights the critical role of intermediate adsorption energy on electrocatalysts in the reaction process. According to the Sabatier principle, which governs heterogeneous electrocatalysis, ideal catalysts must exhibit balanced adsorption energy for reactants and intermediates during electrocatalysis.^[^ ^67^ ^]^ Over‐adsorption prevents the desorption of intermediates and products, whereas under‐adsorption impedes the chemical bonding of reactants, both causing sluggish electrocatalytic kinetics. Thus, the Sabatier principle can be effectively summarized using the adsorption energy of a crucial intermediate as a descriptor of activity. Volcano plots are utilized to identify promising electrocatalysts for EAS, with the applied potential for the eN_2_RR represented as a function of Δ*E*_N*_ (the adsorption energy of the N atom on the electrocatalyst).^[^ ^68^, ^69^ ^]^ The adsorption energy of N_2_H* can also function as an activity descriptor for eN_2_RR. By integrating it with the activity descriptor for the HER, the selectivity of eN_2_RR can be evaluated based on the Δ*G* difference between H* and N_2_H* (Δ*G*_H*_ − Δ*G*_N2H*_). When Δ*G*_H*_ − Δ*G*_N2H*_ is greater than 0, it indicates that the catalyst is favorable for N_2_* hydrogenation, signifying favorable selectivity.^[^ ^70^ ^]^

From an orbital viewpoint, the adsorption of adsorbates on electrocatalyst surfaces can be interpreted as the interaction between the orbitals of the adsorbates and those of the electrocatalyst.^[^ ^71^ ^]^ The filling of electrons into high‐energy antibonding states decreases stability, which is detrimental to adsorption. When it comes to transition metals (TMs), the *s* and *p* orbitals exhibit broad, overlapping shapes, while the d orbitals remain localized. It is commonly acknowledged that the interaction between the adsorbate orbitals and the *s* or *p* orbitals of TMs is generally consistent.^[^ ^72^ ^]^ As a result, the electronic states of the *d*‐band are crucial in determining adsorption behavior, forming the foundation of the well‐known *d*‐band theory.^[^ ^73^ ^]^ This theory has been widely utilized to predict the electrocatalytic activity of various catalysts.^[^ ^74^ ^]^ In *d*‐band theory, the *d*‐band center, a vital descriptor, represents the average state of *d*‐orbital electrons. Stronger bonding between electrocatalysts and adsorbates is linked to higher antibonding energy levels (less likely to be occupied by electrons), which are associated with a *d*‐band center closer to the Fermi level. Moreover, additional descriptors, such as *d*‐band width,^[^ ^75^ ^]^ *d*‐orbital charge,^[^ ^76^ ^]^ and the *p*‐band center for nonmetallic materials,^[^ ^77^ ^]^ have been introduced to further explain adsorption states.

Despite its utility in characterizing the average state of *d*‐orbital electrons, *d*‐band theory faces constraints when utilized for magnetic materials with a substantial number of unpaired electrons, such as TM oxides.^[^ ^78^ ^]^ Thus, recognizing the role of electron spin states and refining the electronic depiction of *d*‐orbitals becomes crucial. In ligand field theory, the interaction between TMs and ligands (nonmetal atoms or adsorbates) causes *d*‐orbital splitting.^[^ ^79^ ^]^ For instance, in an octahedral structure, *d*‐electrons closer to the ligands possess higher energies than those farther away, resulting in the splitting of *d*‐orbitals into low‐energy *t*_2g_ orbitals (*d*_xy_, *d*_xz_, and *d*_yz_) and high‐energy *e*_g_ orbitals (*d*_z_^2^ and *d*_x_^2^‐_y_^2^). This splitting, along with the electron pairing energy, can lead to different spin states, such as high spin, intermediate spin, and low spin. These spin states significantly influence charge transfer and adsorption behavior. For example, high spin states often result in more unpaired electrons, enhancing electrical conductivity. The electrocatalyst exhibits optimal catalytic performance and adsorption capability for key intermediates when the electron filling number in the *e*_g_ orbital is ≈1.^[^ ^80^ ^]^

**Figure 6:**
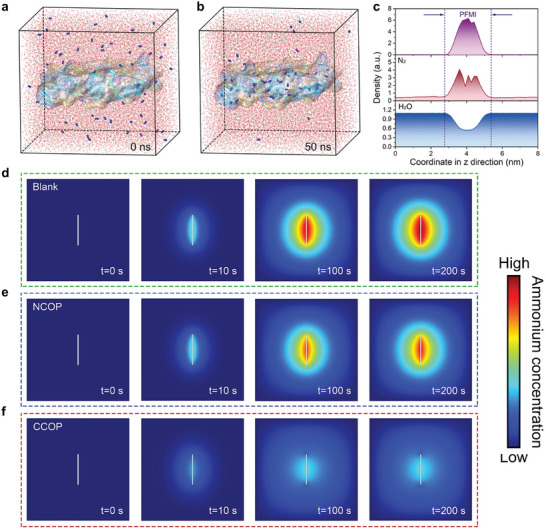
MD snapshots illustrating nitrogen distribution at a) 0 ns and b) 50 ns. Gray lines represent H, red lines denote O, blue spheres indicate N, and the colored isosurface corresponds to the PFMI model. c) Spatial distribution profiles of PFMI, N_2_, and H_2_O along the *z*‐axis within the simulation box at 50 ns. Reproduced with permission.^[^ ^84^ ^]^ Copyright 2023, Wiley‐VCH. NH_3_ distribution profiles at 0, 10, 100, and 200 s within the d) blank, e) neutral covalent organic polymer (NCOP)‐containing, and f) covalent organic polymer with intrinsic cationic sites (CCOP)‐containing models in finite element simulations. Reproduced with permission.^[^ ^85^ ^]^ Copyright 2023, Wiley‐VCH.

Building upon the comprehensive discourse regarding the application of DFT calculations in catalyst design, focusing on the regulation of electronic structures and *d*‐band centers, we now shift our attention to the advancements in molecular dynamics (MD) simulations and COMSOL Multiphysics in catalytic design.^[^ ^81^, ^82^, ^83^ ^]^ MD simulations offer profound insights into the atomic‐level dynamics of catalysts under varying conditions, such as surface reconstruction and the evolution of reaction pathways. Concurrently, COMSOL facilitates the simulation of multiphysics coupling effects in catalysts during operation, encompassing thermal conductivity, fluid dynamics, and electrochemical reactions. The synergy of these methodologies not only enhances our comprehension of catalyst performance but also provides a robust theoretical framework for the design and optimization of catalysts. For instance, Liu et al.^[^ ^84^ ^]^ improved the performance of eN_2_RR catalysts through molecular imprinting, which creates a nitrogen‐rich perfluorinated molecularly imprinted polymer (PFMI) adlayer on the catalyst surface. This approach selectively concentrates nitrogen molecules while restricting water access, thereby tripling the NH_3_ production rate and FE to 185.7 µg h^−1^ mg^−1^ and 72.9%, respectively, for a metal‐free catalyst. MD simulations validated the efficacy of adlayer in sustaining high nitrogen concentrations (65%−84%) and diminishing water content to ≈11%, attributing this to robust nitrogen adsorption and hydrophobic fluorocarbon chains (**Figure** **6**a–c). Similarly, Chen et al.^[^ ^85^ ^]^ developed a novel covalent organic polymer featuring ordered periodic cationic sites to enhance eN_2_RR performance. The strong positive charge of polymer repels ammonium ions, maintaining an ultralow interfacial product concentration and thereby propelling the reaction forward. This strategy, when combined with a transition metal catalyst, led to a 24‐fold increase in FE, reaching 73.74%. COMSOL Multiphysics simulations confirmed the capability of the polymer to facilitate ultrarapid ammonium transfer (100–200 s) due to potent electrostatic interactions, whereas the noncationic polymer showed minimal improvement (Figure 6d–f).

By employing DFT calculations, MD simulations, and COMSOL computations, we have scrutinized catalyst electronic structures, *d*‐band centers, and catalytic performance. These methodologies not only elucidate reaction mechanisms but also guide experimental design, thereby substantially enhancing the efficiency of catalyst development. However, challenges persist, such as computational expenses and parameter selection. The subsequent section will delve into catalyst design strategies informed by these theoretical frameworks, integrating theory with experimentation to optimize catalyst performance.

## 4 Strategies to Improve EAS Performance

In recent years, various electrocatalysts for the eN_2_RR,^[^ ^86^, ^87^, ^88^, ^89^, ^90^, ^91^, ^92^, ^93^, ^94^, ^95^, ^96^, ^97^, ^98^, ^99^, ^100^, ^101^, ^102^, ^103^, ^104^, ^105^, ^106^, ^107^ ^]^ eNO_3_RR,^[^ ^108^, ^109^, ^110^, ^111^, ^112^, ^113^, ^114^, ^115^, ^116^, ^117^, ^118^, ^119^, ^120^, ^121^, ^122^, ^123^, ^124^, ^125^, ^126^, ^127^, ^128^, ^129^, ^130^, ^131^, ^132^, ^133^, ^134^, ^135^, ^136^ ^]^ and eNORR^[^ ^137^, ^138^, ^139^, ^140^, ^141^, ^142^ ^]^ have been developed through vacancy engineering, crystal facet control, hybridization, phase engineering, and other advanced strategies. The subsequent sections will detail the methodologies employed to improve NH_3_ production rates and FEs, alongside an overview of the performance of selected catalysts.

### 4.1 Vacancy Engineering

In the context of aqueous solutions, eN_2_RR is a highly complex three‐phase reaction involving solid catalysts, liquid electrolytes, and gaseous reactants (N_2_). The surface structure and properties of the catalyst play a crucial role in modulating the eN_2_RR performance. To achieve specific surface properties required for optimum performance, the regulation of vacancies in catalysts is commonly utilized. These vacancies can alter the local coordination environment of the electrocatalyst and thus influence its eN_2_RR performance. Surface vacancies at the atomic scale have been extensively studied in nanostructured electrocatalysts for eN_2_RR.^[^ ^143^ ^]^ Due to the presence of abundant localized electrons, vacancies can serve not only as anchoring sites for N atoms but also as active sites that participate in the reaction to enhance N_2_ bond cleavage.^[^ ^144^, ^145^, ^146^ ^]^ For instance, the construction of vacancies, such as oxygen vacancies (OVs),^[^ ^86^, ^87^, ^88^, ^147^, ^148^, ^149^, ^150^, ^151^ ^]^ sulfur vacancies (SVs),^[^ ^92^ ^]^ and nitrogen vacancies (NVs),^[^ ^89^, ^90^, ^91^ ^]^ can effectively generate electron‐deficient sites/regions on the electrocatalysts (typically covering the TM‐based materials), boosting the activation of N_2_ molecules through σ‐donation effects. The distributions of electron‐deficient sites on vacancy‐enriched electrocatalysts are usually dominated by the types of vacancies. In particular, for electrocatalysts with abundant OVs, the electron‐deficient sites are the TM atom sites that are adjacent to the vacancies due to the strong electron‐capture ability of these vacancies. However, NVs are generally the electron‐deficient sites on NV‐enriched electrocatalysts. Since current studies mainly focus on OVs, SVs, and NVs, here, the effects of OVs, SVs, and NVs on activating N_2_ molecules are discussed at length.

Metal oxides are commonly investigated as eN_2_RR catalysts due to their weak HER ability. The construction of OVs on metal oxide surfaces can enhance electron transfer, and regulate the local electronegativity and coordination environment, thereby boosting the eN_2_RR activity.^[^ ^147^, ^148^, ^149^, ^150^, ^151^ ^]^ For instance, Yang et al.^[^ ^86^ ^]^ demonstrated a straightforward and mild amylum‐protection strategy to generate OVs, with the goal of addressing the trade‐off between N_2_ adsorption and NH_3_ desorption. This objective was achieved by constructing a hollow shell structure of Fe_3_C/Fe_3_O_4_ heterojunction coated with a carbon framework (Fe_3_C/Fe_3_O_4_@C). In this heterostructure, the formation of OVs in the Fe_3_O_4_ component was triggered by Fe_3_C. The generated OVs facilitate the generation of electron‐rich catalytic sites and effectively weaken the N≡N for activating N_2_. DFT calculations reveal that the Δ*G*_max_ for converting N_2_ to NH_3_ on Fe_3_C/Fe_3_O_4_ and the oxygen‐vacancy‐rich Fe_3_C/Fe_3_O_4_ (O‐Fe_3_C/Fe_3_O_4_) are 0.93 and 0.83 eV, respectively, indicating that O‐Fe_3_C/Fe_3_O_4_ is thermodynamically more favorable. Specifically, while N_2_ adsorption on Fe_3_C/Fe_3_O_4_ is exothermic and facilitates N_2_ activation into N_2_H*, the formation of NH_2_‐NH_2_* species requires overcoming a 0.93 eV energy barrier. Moreover, the unbalanced adsorption strength of N*_x_* H*_y_* intermediates on Fe_3_C/Fe_3_O_4_ results in a higher energy requirement (Δ*G* = 2.16 eV) for NH_3_ desorption. In contrast, O‐Fe_3_C/Fe_3_O_4_ effectively balances the adsorption strengths of N_2_ and N*_x_* H*_y_* intermediates. Ultimately, the adsorption strength of both N_2_ and N*_x_* H*_y_* intermediates was optimized, thereby the eN_2_RR activity was augmented.

**Figure 7:**
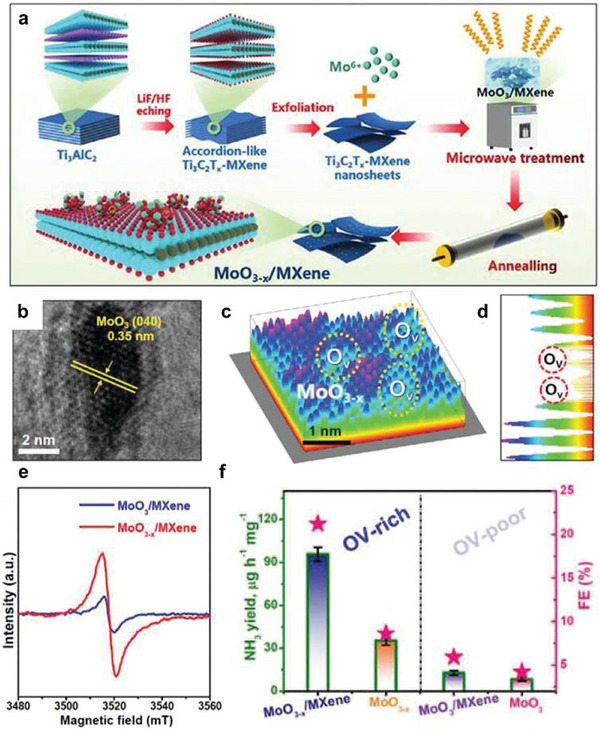
a) Schematic representation of the synthesis process for MoO_3−_ *_x_*/MXene. b) High‐resolution transmission electron microscope (HRTEM) image. c) 3D atom imaging of a MoO_3−_ *_x_* nanoparticle on MXene and corresponding lattice line scanning d). e) Electron paramagnetic resonance (EPR) spectra of MoO_3_/MXene and MoO_3−_ *_x_*/MXene. f) NH_3_ yield rates and FEs of MoO_3_, MoO_3−_ *_x_*, MoO_3_/MXene, and MoO_3−_ *_x_*/MXene. Reproduced with permission.^[^ ^88^ ^]^ Copyright 2022, Wiley‐VCH.

Regulation of N_2_ adsorption can also be achieved using modulation of the interaction between the OV‐rich oxide and the carrier. Take vacancy‐enriched MoO_3−_ *_x_* anchored on Ti_3_C_2_T*_x_*‐MXene (MoO_3−_ *_x_*/MXene) as an example (**Figure** **7**), this electrocatalyst achieves a remarkable NH_3_ yield rate of 95.8 µg h^−1^ mg^−1^ and a FE of 22.3%.^[^ ^88^ ^]^ Integration of in situ spectroscopy, MD simulations, and DFT calculation was used to unveil the synergistic effect of vacancies and heterostructures in eN_2_RR. The MD results demonstrate significant N_2_ enrichment at the OV‐site, whereas no notable N_2_ accumulation is observed on OV‐poor MoO_3_/Mxene. The results demonstrated that the OV sites on MoO_3−_ *_x_* served as the active sites for N_2_ chemisorption and activation, while the MXene substrate regulated these OV sites to break scaling relations and stabilize *N_2_/*N_2_H effectively while destabilizing *NH_2_/*NH_3_, resulting in more optimized binding affinity of eN_2_RR intermediates toward reduced energy barriers and an enhanced eN_2_RR activity for MoO_3−_ *_x_*/MXene.

Alternatively, OVs can also be induced by grain boundary engineering.^[^ ^87^ ^]^ For example, Zhong et al. developed an eN_2_RR catalyst through in situ anchoring of interfacial intergrown ultrafine MoO_2_ nanograins on N‐doped carbon fibers. The proportion of OVs could be controlled by regulating the grain boundaries between MoO_2_ nanograins through optimizing the thermal treatment conditions, thereby enhancing the transfer of electrons, yielding highly active reactive sites and efficient nitrogen trapping. The optimal catalyst, referred to as MoO_2_/C700, exceeded the performance of commercially available MoO_2_ and state‐of‐the‐art catalysts for eN_2_RR, with a yield rate of NH_3_ and FE of 173.7 µg h^−1^ mg^−1^_cat_ and 27.6%, respectively, at −0.7 V versus RHE in a 1 m KOH electrolyte solution. The in situ X‐ray photoelectron spectroscopy (XPS) and DFT calculation validated the electronic structure effect and advantage of N_2_ adsorption over OVs, revealing the dominant interplay between N_2_ and OVs. Due to the similar chemical properties of oxygen and sulfur, introducing SVs in sulfur‐containing catalysts is expected to enhance the eN_2_RR performance. Zi et al.^[^ ^92^ ^]^ designed an SV‐rich single‐layered 1T‐MoS_2_ which is uniformly grown on the MoO_3_ matrix (denoted as SV‐1T‐MoS_2_@MoO_3_) as eN_2_RR catalyst via an interfacial engineering strategy. Benefiting from the functional SVs, well‐designed structure, and comparative advantages of the metallic 1T‐MoS_2_ phase, the SV‐1T‐MoS_2_@MoO_3_ electrocatalyst exhibits superior eN_2_RR performance than some other MoS_2_‐based counterparts in acid electrolyte. DFT calculations showed that the SVs in SV‐1T‐MoS_2_@MoO_3_ could regulate its electronic structure and move the antibonding 2π* orbital of the N_2_ molecule closer to the Fermi level, leading to a more favorable direction for eN_2_RR. Electrocatalysts with NVs, such as polymeric carbon nitride,^[^ ^89^ ^]^ 2D layered W_2_N_3_ nanosheet,^[^ ^90^ ^]^ and P doping in C_3_N_4_^[^ ^91^ ^]^ have been developed for eN_2_RR. For example, due to the high valence state of W atoms and the 2D confinement effect, NVs can stably exist on the surface of the 2D W_2_N_3_ nanosheet. The NVs on W_2_N_3_ provide an electron‐deficient environment, facilitating the adsorption of nitrogen and lowering the thermodynamic limiting potential of eN_2_RR. Consequently, the nanosheets exhibit a consistent NH_3_ yield rate of 11.66 ± 0.98 µg h^−1^ mg^−1^, with an FE of 11.67 ± 0.93%.

**Figure 8:**
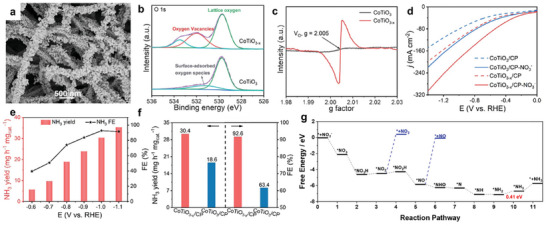
a) Scanning electron microscopy images of CoTiO_3−_ *_x_*. b) XPS spectra of CoTiO_3_ and CoTiO_3−_ *_x_* in O 1s region. c) EPR spectra of CoTiO_3_ and CoTiO_3−_ *_x_*. d) Linear scanning voltammetry (LSV) curves of CoTiO_3−_ *_x_*/carbon paper and CoTiO_3_/CP in 0.1 m NaOH with and without 0.1 m NO_3_^−^. e) NH_3_ yield rates and FEs of CoTiO_3−_ *_x_*/CP at various applied potentials. f) NH_3_ yield rates and FEs of CoTiO_3−_ *_x_*/CP and CoTiO_3_/CP at −1.0 V. g) Free energy diagrams for eNO_3_RR on the CoTiO_3_ (104) surface with V_2O1_. Reproduced with permission.^[^ ^129^ ^]^ Copyright 2022, Wiley‐VCH.

In addition to eN_2_RR, vacancy engineering is also effective in improving the intrinsic activity of eNO_3_RR. Especially it could also suppress the side reactions, including the formation of other products, such as N_2_, N_2_O, NO, and NO_2_. Jia et al.^[^ ^49^ ^]^ reported on TiO_2−_ *_x_* nanotubes that contain rich OVs and exhibit both high FE (85.0%) and selectivity (87.1%) in NH_3_ synthesis from eNO_3_RR. OVs in TiO_2−_ *_x_* are found to play a critical role in eNO_3_RR through online differential electrochemical mass spectrometry (DEMS) and DFT calculations. Specifically, the oxygen atom in NO_3_^−^ fills in the vacancies of TiO_2−_ *_x_* to weaken the N*─*O bonding and suppress the formation of byproducts, leading to high FE and NH_3_ selectivity. The research conducted by Fan et al.**^[^** ^129^ ^]^ has experimentally demonstrated that CoTiO_3−_ *_x_* nanofibers containing OVs serve as highly efficient catalysts for the EAS through eNO_3_RR (**Figure** **8**). The CoTiO_3−_ *_x_*/CP electrode exhibits significantly improved performance compared to the CoTiO_3_/CP electrode under ambient conditions. It achieves an NH_3_ yield rate of 30.4 mg h^−1^ mg_cat._^−1^ and a high FE of 92.6%, whereas the CoTiO_3_/CP electrode yields 18.6 mg h^−1^ mg_cat_.^−1^ with an FE of 63.4%. Furthermore, a Zn‐NO_3_^−^ battery employing the CoTiO_3−_ *_x_*/CP cathode demonstrates a power density of 5.04 mW cm ^−2^ and an NH_3_ yield rate of 3.08 mg h ^−1^ mg_cat._^−1^. DFT calculations suggest that the OVs on the CoTiO_3_ (104) surface favor the adsorption of NO_3_^−^. Additionally, the *NH_2_ hydrogenation to *NH_3_ is identified as the rate‐determining step (RDS) with a favorable free energy change (Δ*G*) of 0.41 eV, which is significantly lower than that of pristine CoTiO_3_ (104) with a Δ*G* of 1.15 eV. Dong et al.^[^ ^130^ ^]^ developed a method to generate and systematically control OVs in ZnCr_2_O_4_ nanofibers by substituting chromium ions with zinc. This enrichment of zinc induces the formation of zero‐valent zinc and effectively modulates the OV in the spinel ZnCr_2_O_4_ nanofiber. The resulting defective fiber catalyst exhibits activity for eNO_3_RR, with an NH_3_ yield rate of 20.36 mg h^−1^ mg^−1^_cat._ and a FE of 90.21%. Theoretical simulations reveal the large NO_3_^−^ adsorption energy and low desorption energy of NH_3_ at the catalyst surfaces, thus synergistically enhancing the performance. Du et al.^[^ ^117^ ^]^ propose an OV‐rich pseudobrookite Fe_2_TiO_5_ nanofiber as an electrocatalyst for the ambient eNO_3_RR. This catalyst achieves an NH_3_ yield rate of 0.73 mmol h^−1^ mg^−1^_cat._ and a FE of 87.6% in a phosphate buffer saline solution containing 0.1 m NaNO_3_. The NH_3_ yield rate is elevated to 1.36 mmol h^−1^ mg^−1^_cat._ and the FE reaches 96.06% for the conversion of nitrite to NH_3_ in 0.1 m NaNO_2_. Furthermore, DFT calculations reveal the formation of antibonding states with the binding of Fe and O that lowers the Fermi energy and promotes charge transfer, and meanwhile, OV shifting the *d*‐band center to a higher level supports the boosted catalytic activity. Besides OVs, the other type of vacancy can also facilitate the eNO_3_RR. For example, Gao et al.^[^ ^131^ ^]^ constructed a heterogeneous bimetallic phosphide CoP‐Ni_2_P with controllable phosphorus vacancies (PVs) by utilizing the doping‐oxygen strategy. Theoretical calculations indicate that the introduction of PVs accelerates the RDS of eNO_3_RR, thus enhancing the reaction kinetics. Consequently, the conversion, FE, and selectivity of the catalyst are substantially enhanced. Furthermore, the Zn‐NO_3_^−^ battery demonstrated a power density of 1.05 mW cm^−2^.

The single atomic vacancies (SAVs) also exert a certain influence on the coordination environment. Based on the high‐throughput first‐principles calculation, He et al.^[^ ^152^ ^]^ theoretically demonstrate that TM‐doped MoS_2_ (TM@MoS_2_) with the sulfur SAVs (TM@MoS*_v_*) can achieve high selectivity for the conversion of NO to NH_3_ due to the spatial confinement effect. Furthermore, aliovalent ion doping can optimize eNORR activity by modifying the polarization charge distribution of active sites and effectively altering the intermediate binding strength. As a result, six active, selective, and stable eNORR catalysts were identified, including La‐doped MoS_2_ with a sulfur SAV, which exhibits a low limiting potential of −0.15 V. Importantly, the formation energy of the sulfur SAV is identified as an intrinsic descriptor for the design of eNORR catalysts. The EAS performances of partial electrocatalysts enhanced by vacancy engineering are listed in Table 1.

### 4.2 Crystal Facet Design

The crystal orientation and crystallization of the catalyst play a vital role in eN_2_RR. Specifically, selectively exposing certain crystal facets can optimize the RDS in eN_2_RR, thereby improving its activity. For example, Zhao et al.^[^ ^93^ ^]^ conducted a systematic investigation of Pd nanocrystals with selectively exposed (100), (111), and (110) facets, each of which exhibits cubic, octahedral, and rhombic dodecahedral morphology, respectively. Pd cubes display an NH_3_ yield rate of 24.3 µg mg^−1^_cat_ h^−1^ and a FE of 36.6%. These values were 2.7 and 5.3 times higher, respectively, than those observed for Pd octahedrons and Pd rhombic dodecahedrons. DFT calculations revealed that Pd (100) has remarkable eN_2_RR performance, which can be attributed to the lower energy barrier for generating *NNH and the lower energy barrier for producing NH_3_ from *NH_3_ (RDS). There are also some more examples of eN_2_RR activity enhancement achieved through crystal‐facet engineering. For instance, Yang et al.^[^ ^153^ ^]^ found that the catalytic activity of (110)‐oriented Mo was better than that of (211) orientation. Additionally, Abghoui and Skúlason^[^ ^154^ ^]^ demonstrated that the (111) planes of TiN, VN, CrN, MnN, ZrN, NbN, MoN, HfN, WN, and ReN are effective for eN_2_RR. Through theoretical analysis, it was discovered that nitride crystal faces exhibit strong selectivity in the eN_2_RR. These findings further confirm that eN_2_RR is a surface‐sensitive process, bringing attention to the significance of adjusting the crystal orientation in catalyst design. Furthermore, high‐index facet engineering also influences the electronic structure of active sites, thereby enhancing the performance of eN_2_RR. Tong et al.^[^ ^94^ ^]^ report the controlled synthesis of well‐defined Pt_3_Fe nanoparticles with adjustable morphologies (nanocube, nanorod, and nanowire) as ideal electrocatalyst models for investigating eN_2_RR on different exposed facets. The detailed electrocatalysis studies reveal that the Pt_3_Fe nanoparticles exhibit shape‐dependent electrocatalysis of eN_2_RR. The optimized Pt_3_Fe nanowires bounded by high‐indexed facets exhibit higher NH_3_ yield rate and selectivity toward eN_2_RR, outperforming {200} facet‐enclosed Pt_3_Fe nanocubes and {111} Pt_3_Fe nanorods. DFT calculations reveal that, with high‐indexed facet engineering, the Fe‐3d band serves as an efficient correlation center for *d*–*d* coupling, boosting the exchange and transfer activities of Pt 5d‐electrons toward eN_2_RR.

The crystal‐facet engineering has become a promising strategy for enhancing catalytic activity through atomic‐scale manipulation of the surface structures of catalysts. Meanwhile, it will also influence the surface vacancies (such as OVs), the electronic structure of active sites, and energy barriers to the RDS. Recently, Zhong et al.^[^ ^155^ ^]^ have manipulated the surface oxygen species of Cu_2_O through facet engineering, conducting a systematic investigation into the influence of these oxygen species on the activity of eNO_3_RR. The OVs on the Cu_2_O (111) surface will enhance the adsorption of reactants and reaction intermediates. Additionally, hydroxyl groups effectively suppress the side reaction of HER and promote the hydrogenation process of eNO_3_RR. These two effects synergistically contribute to making the Cu_2_O (111) facet exhibit the highest eNO_3_RR activity compared to other facets. There are some other examples of surface defects that have been modulated using the crystal‐facet design strategy, Hu et al.^[^ ^109^ ^]^ demonstrated an electrocatalyst synthesis strategy based on the in situ electrochemical reduction of ultrathin copper‐oxide nanobelt under eNO_3_RR conditions. The reduction method exposes Cu (100) facets and abundant surface defects, which favorably facilitates the eNO_3_RR while hindering the HER. It was found that the nitrogen species (N*) produced during eNO_3_RR function as capping agents that control the exposed facets during the reduction. Remarkably, in alkaline media, the eNO_3_RR catalyzed by defective Cu (100) facets gives an NH_3_ yield rate that is 2.3‐fold higher than that of the H–B process. The synergy of Cu (100) facets and defects, which upshift the *d* band center of Cu, is key to the excellent performance of the electrocatalyst. The eNO_3_RR activity can also be enhanced by the regulation of nanocatalysts with controlled facets, such as nanocubes, cuboctahedrons, octahedrons, and concave nanocubes. Lim et al.^[^ ^156^ ^]^ demonstrated that, in an alkaline electrolyte, Pd(111) > Pd(100) > Pd(hkl) for NO_3_^−^ reduction activity and Pd(100) > Pd(hkl) > Pd(111) for nitrite reduction activity. Cuboctahedrons with both Pd (111) and Pd (100) facets demonstrated the highest production of NH_3_ (306.8 µg h^−1^ mg_Pd_^−1^) with a FE of 35%. On the other hand, nanocubes that only expose Pd (100) showed high activity in reducing NO_2_^−^ to NH_3_, while octahedrons without Pd (100) facets produced only NO_2_^−^, and negligible amounts of NH_3_ and N_2_. DFT simulations showed that *NO_3_ dissociation to *NO_2_ + *O is more favorable on Pd (111) facets than Pd (100) facets, which explains why there is a faster NO_3_^−^ reduction kinetics on Pd (111) facet observed in the experiments. Additionally, *NO_2_ binds less strongly to Pd (111) compared to Pd (100), which means that NO_2_^−^ formed via NO_3_^−^ dissociation readily desorbs from the Pd (111) surface, explaining why Pd (111) selectively reduces NO_3_^−^ to NO_2_^−^. The results show that cuboctahedrons are bifunctional in nature, where the (111) facet catalyzes the conversion of NO_3_^−^ to NO_2_^−^ and the (100) facet catalyzes the conversion of NO_2_^−^ to NH_3_. In addition to the above by controlling a single specific crystalline surface, the utilization of the multicrystalline surface synergistic effect can also promote the eNO_3_RR performance. A flower‐like open‐structured polycrystalline copper (FOSP‐Cu), loaded onto carbon fiber papers, has been successfully prepared by linearly varying the deposition potential.^[^ ^157^ ^]^ This approach allows for the continuous growth of different Cu crystal planes (such as Cu (100) and Cu (111)), which facilitates synergistic catalysis during the 8‐electron eNO_3_RR. The optimal sample achieves an NH_3_ yield rate of 101.4 µmol h^−1^ cm^−2^ and an FE of 93.91% in a neutral solution.

A hexagonal close‐packed (hcp) Co nanosheet was prepared by Wang et al.^[^ ^139^ ^]^ and exhibited a NH_3_ yield rate of 439.50 *µ*mol cm^−2^ h^−1^ and a FE of 72.58%. It outperforms the face‐centered cubic phase (fcc) of the Co nanosheet (NH_3_ yield rate, 142.1 *µ*mol h^−1^ cm^−2^; FE, 57.12%). The combination of DFT calculations and NO temperature‐programmed desorption experiments reveals that the hcp‐Co shows superior eNORR activity due to its unique electron structures and proton shuttle effect. A device of Zn‐NO batteries using the hcp‐Co as the cathode is assembled, demonstrating a power density of 4.66 mW cm^−2^. Zhao et al^[^ ^158^ ^]^ screened a series of TMs (Ti, V, Cu, Ni, Pd, Ag, and Au) to investigate the relationship between the electronic structure and product distribution in eNORR. Among these TMs, Ni outperformed others with an NH_3_ yield rate of 9.48 *µ*mol cm^−2^ h^−1^. Meanwhile, five monocrystalline Ni foils with various surface orientations were designed and prepared. And Ni with high‐index facets exhibited higher eNORR activity. In particular, Ni (210) demonstrates 100% selectivity of NH_3_ with a yield rate of 12.02 *µ*mol cm^−2^ h^−1^. DFT calculations indicate that the abundant steps of high‐index facets promote a decrease in the energy required for key intermediates associated with the RDS hydrogenation step. In a flow cell, electrochemical measurements on Ni nanoparticle ensembles demonstrated an NH_3_ selectivity of over 85% with a yield rate of 544 *µ*mol cm^−2^ h^−1^, with stable operation over 50 h. The EAS performances of partial electrocatalysts enhanced by crystal facet design are listed in Table 2.

### 4.3 Hybridization Engineering

**Figure 9:**
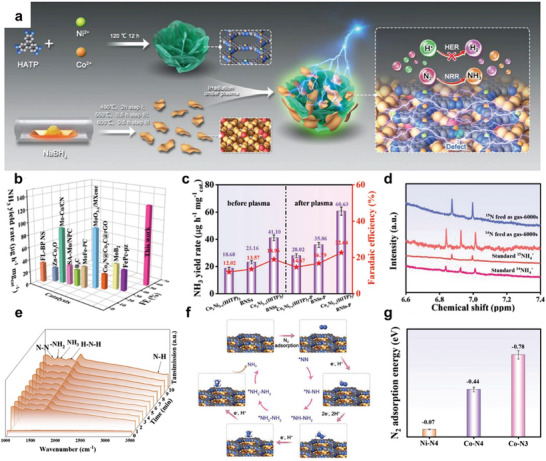
a) Schematic depicting the fabrication process of Co*_x_* Ni_3−_ *_x_* (HITP)_2_/BNSs‐P for eN_2_RR. b) Comparison of the NH_3_ yield rate of various eN_2_RR catalysts in 0.1 m HCl. c) Performance evaluation of different catalysts for eN_2_RR. d) Isotope labeling experiments using ^15^N_2_ and ^14^N_2_. e) Electrochemical in situ Fourier transform infrared spectroscopy (FTIR) spectra of Co*_x_* Ni_3−_ *_x_* (HITP)_2_/BNSs‐P during eN_2_RR. f) Mechanistic insights into the eN_2_RR on Co*_x_* Ni_3−_ *_x_* (HITP)_2_/BNSs‐P. g) Calculated N_2_ adsorption energies for Ni‐N_4_, Co‐N_4_, and Co‐N_3_ active sites. Reproduced with permission.^[95]^ Copyright 2023, Wiley‐VCH.

In the field of catalysis, hybridization plays a crucial role in enhancing the catalytic activity of materials. It improves the activity by adjusting the electronic structure and creating additional active sites at the interface between different components. At the interface of different components, electrons are transferred and form the electron–hole pairs that can take part in surface reactions. As a result, this method has been extensively studied in eN_2_RR. Liu et al.^[^ ^95^ ^]^ have designed an effective *p*‐*n* heterojunction of semiconductive metal‐organic framework (MOF) Co*_x_* Ni_3−_ *_x_* (HITP)_2_ and boron nanosheets (BNSs), named Co*_x_* Ni_3−_ *_x_* (HITP)_2_/BNSs‐P, through in situ solution plasma modification (**Figure** **9**). Under plasma treatment, the work function of the catalyst is significantly lowered, and the active energy of N_2_ molecules is decreased due to the formation of the heterojunction at the interface between the ultrathin MOF and BNSs. By introducing a second metal ion into the MOFs skeletons, more unsaturated metal‐coordination sites can be generated, thus modulating the electron density of the active sites and enhancing catalytic activity. Interface engineering and plasma‐assisted defects on the Co*_x_* Ni_3−_ *_x_* (HITP)_2_/BNSs‐P heterojunction led to the formation of both Co‐N_3_ and B…O dual‐active sites. Therefore, Co*_x_* Ni_3−_ *_x_* (HITP)_2_/BNSs‐P exhibited a higher NH_3_ yield rate (128.26 ± 2.27 µg h^−1^ mg_cat._^−1^) and a FE (52.92 ± 1.83%), which significantly exceeds the performance of the individual components.

Furthermore, inspired by the structure of nitrogenase in nature, catalysts designed through hybridization engineering also possess eN_2_RR activity. For example, Li et al.^[^ ^96^ ^]^ have rationally designed a bio‐inspired electrocatalyst of NiCoP/CoMoP/Co(Mo_3_Se_4_)_4_@C/NF, which simulates the structural characteristics of biological nitrogenase. Namely, they form triple heterojunction interfaces during synthesis: NiCoP‐CoMoP, NiCoP‐Co(Mo_3_Se_4_)_4_, and NiCoP‐Co(Mo_3_Se_4_)_4_. These coordinate to extend the catalytic sites and optimize the reaction energy barrier of the intermediates. DFT calculations reveal that CoMoP can be used as an electron carrier to transfer electrons from NiCoP to Co(Mo_3_Se_4_)_4_. This results in the accumulation of electrons in Co(Mo_3_Se_4_)_4_, significantly lowering the energy barrier for the first hydrogenation of eN_2_RR. Furthermore, the electron redistribution caused by the coupling of the triple heterojunctions enhances the electrical conductivity and interconnectivity for efficient charge transfer, resulting in optimized adsorption behavior of intermediates. Consequently, the NiCoP/CoMoP/Co(Mo_3_Se_4_)_4_@C/NF multiheterojunction nanoflowers exhibit eN_2_RR performance with an NH_3_ yield rate of 24.54 µg h^−1^ cm^−2^ and FE of 23.15%. Lv et al.^[^ ^159^ ^]^ developed PdO/Pd heterojunctions supported by carbon nanotubes (PdO/Pd/CNTs) with a controllable mass ratio of Pd to PdO. Compared to PdO/CNTs, PdO/Pd/CNTs exhibit an optimal Pd‐to‐PdO mass ratio of 18%–82% and abundant PdO‐Pd interfaces, which act as active sites for N_2_ activation and proton transitions. This synergistic effect of Pd and PdO shortens the proton transmission route and reduces the overpotential of the chemical reaction.

Moreover, Liang et al.^[^ ^142^ ^]^ developed an amorphous B_2.6_C catalyst supported on a TiO_2_ nanoarray on a Ti plate (a‐B_2.6_C@TiO_2_/Ti) for the eNORR. The a‐B_2.6_C@TiO_2_/Ti demonstrated a NH_3_ yield rate of 3678.6 µg h^−1^ cm^−2^ and a FE of 87.6%, surpassing the performance of TiO_2_/Ti (NH_3_ yield rate, 563.5 µg h^−1^ cm^−2^; FE, 42.6%) and a‐B_2.6_C/Ti (NH_3_ yield rate, 2499.2 µg h^−1^ cm^−2^; FE, 85.6%). The Zn‐NO battery based on an a‐B_2.6_C@TiO_2_/Ti catalyst achieves a power density of 1.7 mW cm^−2^ and an NH_3_ yield rate of 1125 µg h^−1^ cm^−2^. DFT calculations indicate that B*─*C bonding over a‐B_2.6_C layer effectively injects electrons to NO_π2p*_, activating NO and facilitating full reduction with small energy input. Wu et al.^[^ ^160^ ^]^ have designed a Co‐based composite with a heterostructure, which serves as a highly efficient catalyst for eNORR. Additionally, through the integration of B to modulate the electronic structure, the CoB/Co@C catalyst achieved an NH_3_ yield rate of 315.4 µmol h^−1^ cm^−2^, along with a power density of 3.68 mW cm^−2^ in a Zn‐NO battery. The superior eNORR performance of CoB/Co@C can be attributed to the enrichment of NO through cobalt and boron dual‐site adsorption, as well as its fast charge‐transfer kinetics. This demonstrates the pivotal role of boron in enhancing NO enrichment, suppressing HER, and promoting cobalt oxidation, thereby boosting the overall performance of eNORR. Zhang et al.^[^ ^140^ ^]^ reported that MoS_2_ nanosheet on graphite felt (MoS_2_/GF) acts as an efficient and robust 3D electrocatalyst for the conversion of NO to NH_3_. In an acidic electrolyte, such MoS_2_/GF achieves an FE of 76.6% and an NH_3_ yield rate of 99.6 µmol cm^−2^ h^−1^. The assembled Zn‐NO battery device utilizing MoS_2_ nanosheet‐loaded carbon paper as the cathode delivered a discharge power density of 1.04 mW cm^−2^ and an NH_3_ yield rate of 411.8 µg mg^−1^ h^−1^. DFT calculations indicate that the positively charged Mo‐edge sites facilitate NO adsorption and activation via a mechanism of acceptance and donation, while inhibiting proton binding and N*─*N bond coupling. The EAS performances of partial electrocatalysts enhanced by hybridization engineering are listed in Table 3.

### 4.4 Phase Engineering

**Figure 10:**
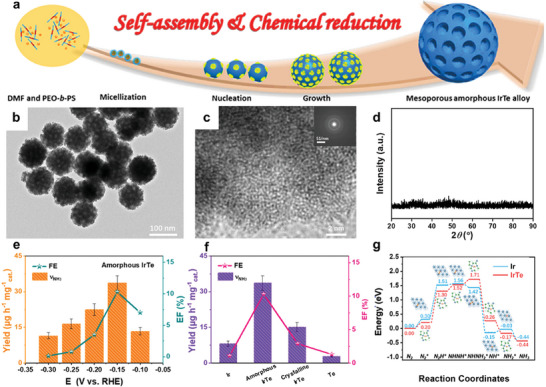
a) Schematic representation of the micelle‐directed bottom‐up synthesis approach for the preparation of mesoporous amorphous IrTe alloy. b) TEM, c) HRTEM, and d) XRD of mesoporous amorphous IrTe alloy nanospheres. e) Histograms of the NH_3_ yield rate and corresponding FEs of mesoporous amorphous IrTe at different applied potentials. f) Comparation of NH_3_ yield rate and FEs at −0.15 V for different catalysts. g) Free energy diagrams depicting the eN_2_RR process on the Ir and IrTe surfaces. Reproduced with permission.^[^ ^98^ ^]^ Copyright 2023, American Chemical Society.

The design of amorphous catalysts in a metastable state with a disordered structure has garnered significant attention for enhancing  intrinsic activity and increasing the number of active sites. Altering crystallinity can lead to diverse atomic arrangements and the introduction of defective sites and under‐coordinated dangling bonds. Consequently, amorphous catalysts typically demonstrate superior catalytic activity compared to their crystalline counterparts due to the higher number of active sites on the defect‐rich surface. For instance, it has been reported that CeO*_x_*‐induced amorphization of Au nanoparticles anchored on the reduced graphite oxide achieves a high NH_3_ yield rate (8.3 µg h^−1^ mg^−1^_cat._) and FE (10.10%), which is higher than the performance of its crystalline counterpart.^[^ ^161^ ^]^ In addition to noble metals, cerium oxide can also be used to regulate the phase engineering of metal oxides, for example, Lv et al. documented the achievability of phase conversion of Bi_4_V_2_O_11_ from crystalline to amorphous by introducing CeO_2_, the presence of localized electrons in the amorphous phase could be enhanced for π‐back donation, thereby contributing to N_2_ activation.^[^ ^162^ ^]^ Subsequently, the team proceeded to develop an amorphous BiNi alloy (a‐BiNi) with a 3D interconnected nanoporous structure to significantly enhance eN_2_RR performance compared to the counterparts of crystalline and pure metal. This improvement is attributed to the chemisorption of nitrogen, lower activation energy after substituting Ni, and the amorphous nature of the alloy framework. Furthermore, additional electrochemical analyses demonstrated improved electron transfer and an increased electrochemical surface area of the 3D nanoporous alloy framework, resulting in the enhanced activity of eN_2_RR. In addition, the interconnected porous structure provides high structural stability for long‐term eN_2_RR, enabling its application in stable and efficient eN_2_RR for potential practical purposes.^[^ ^97^ ^]^ Other alloys containing noble metals can also improve their performance through this strategy. Jiang et al.**^[^** ^98^ ^]^ have reported a mesoporous amorphous noble‐metal alloy, iridium‐tellurium (IrTe), obtained using a micelle‐directed synthesis (**Figure** **10**). The resulting mesoporous amorphous IrTe electrocatalyst displays eN_2_RR activity. The NH_3_ yield rate is 34.6 µg mg^−1^ h^−1^ with a FE of 11.2%, outperforming comparable crystalline and iridium‐metal counterparts. The interconnected porous scaffold and amorphous nature of the alloy create a complementary effect that simultaneously enhances N_2_ absorption while suppressing the HER. According to theoretical simulations, incorporating Te in the IrTe alloy effectively enhances the adsorption of N_2_ and lowers the Δ*G* for the RDS of the eN_2_RR.

**Figure 11:**
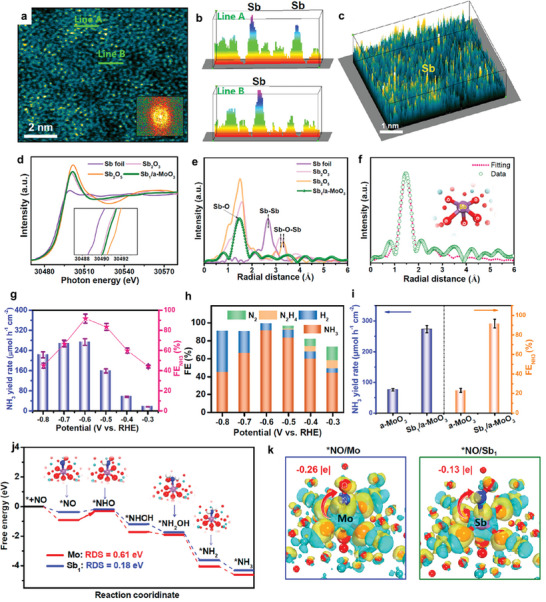
a) The aberration‐corrected high‐angle annular dark‐field scanning transmission microscopy image, accompanied by corresponding b) atom intensity analysis, and c) 3D atom image. d) Sb K‐edge X‐ray absorption near‐edge structure (XANES) and e) extended X‐ray absorption fine structure (EXAFS) spectra of Sb_1_/a‐MoO_3_. f) EXAFS fitting curve of Sb_1_/a‐MoO_3_, with the fitting model shown in the inlet). g) Measured NH_3_ yield rates and corresponding FEs. h) FEs of various products at different potentials. i) Comparison of NH_3_ yield rates and FE NH_3_ of a‐MoO_3_ and Sb_1_/a‐MoO_3_ at −0.6 V. j) Free energy diagrams of alternating‐N pathway on a‐MoO_3_ and Sb_1_/a‐MoO_3_. k) Charge density differences of NO adsorption on a‐MoO_3_ and Sb_1_/a‐MoO_3_. Reproduced with permission.^[^ ^141^ ^]^ Copyright 2023, American Chemical Society.

*p*‐block Sb SAs confined in amorphous MoO_3_ (Sb_1_/a‐MoO_3_) were designed by Chen et al.^[^ ^141^ ^]^ to act as an efficient eNORR catalyst with a NO‐to‐NH_3_ FE of 91.7% and an NH_3_ yield rate of 273.5 µmol cm^−2^ h^−1^ (**Figure** **11**). The results of in situ spectroscopic characterizations and DFT calculations indicate that the eNORR performance of Sb_1_/a‐MoO_3_ arises from the isolated Sb_1_ sites, which can result in optimization of the adsorption of *NO/*NHO to lower the reaction energy barriers and exhibit a higher affinity for NO compared to H_2_O/H species. This strategy can be extended to prepare Bi_1_/a‐MoO_3_, which also shows a high eNORR property.

**Figure 12:**
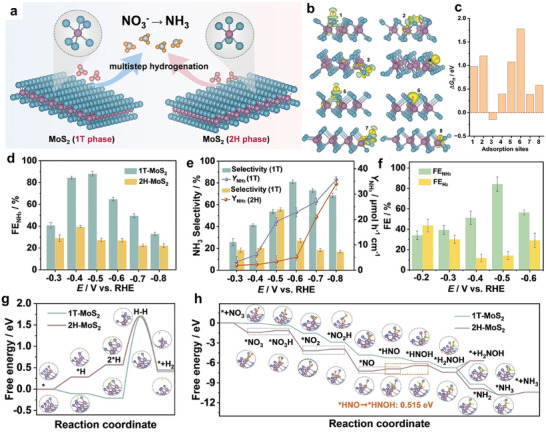
a) Schematic representation of MoS_2_ catalysts exhibiting distinct atomic configurations: octahedron (1T‐MoS_2_) and triangular prism (2H‐MoS_2_) for eNO_3_RR. b) Illustration of charge density differences and c) energies associated with hydrogen adsorption on various sites. d) Potential‐dependent FE of NH_3_ over 1T‐ and 2H‐MoS_2_. e) Potential‐dependent selectivity and yield rate of NH_3_ over 1T‐MoS_2_. f) The FE of generated H_2_ and NH_3_ after eNO_3_RR over 1T‐MoS_2_. g) The reaction energies of H_2_ formation over 1T‐ and 2H‐MoS_2_. h) Free‐energy diagram for NO_3_^−^‐to‐NH_3_ conversion over 1T‐ and 2H‐MoS_2_. Reproduced with permission.^[^ ^163^ ^]^ Copyright 2023, Wiley‐VCH.

Wang et al.**^[^** ^163^ ^]^ used different crystal phases of MoS_2_, including 1T and 2H, as models for an enzyme‐like catalyst (**Figure** **12**). The FE of NH_3_ was ≈90% over 1T‐MoS_2_, clearly outperforming that of 2H‐MoS_2_ (27.31%). In situ Raman spectroscopy and theoretical calculations indicate that 1T‐MoS_2_ generates more active hydrogen on edge S sites at a more positive potential and facilitates a direct pathway from NO_3_^−^ to NH_3_ instead of multiple energetically demanding hydrogenation steps, for example, *HNO to *HNOH, performed on 2H‐MoS_2_. The EAS performances of partial electrocatalysts enhanced by phase engineering are listed in Table 4.

### 4.5 Coordination Environment Modulation

**Figure 13:**
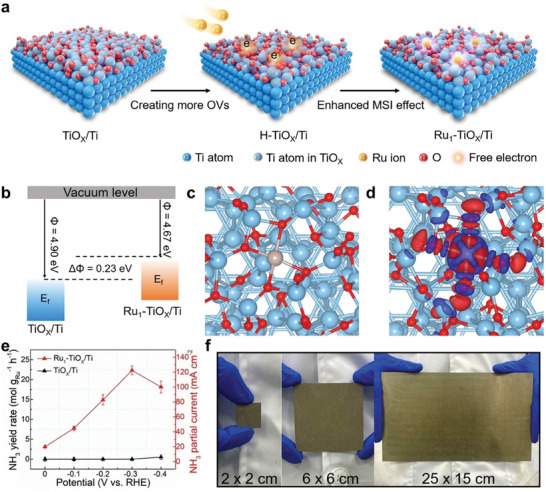
a) Schematic representation of the preparation of Ru_1_–TiO*_x_*/Ti electrode using an inherent surface oxide anchoring strategy. b) Illustration of the work functions for Ru_1_–TiO*_x_*/Ti and TiO*_x_*/Ti, where *E*_f_ represents the Fermi level. c) Structural model of Ru_1_–TiO*_x_*/Ti composite. d) Pots of charge density difference of Ru_1_–TiO*_x_*/Ti. e) NH_3_ yield rate and NH_3_ partial current density at various potentials. f) Pictures of the Ru_1_–TiO*_x_*/Ti electrode in various sizes. Reproduced with permission.^[^ ^134^ ^]^ Copyright 2022, Wiley‐VCH.

The regulation of the coordination environment at the atomic level can enhance the intrinsic activity of eNO_3_RR. For example, Wang et al.^[^ ^164^ ^]^ investigated the eNO_3_RR on TMN_3_ and TMN_4_ (TM = Ti‐Ni) doped graphene using first‐principles calculations. The study found that FeN_4_‐doped graphene shows eNO_3_RR activity with a low limiting potential of −0.38 V, which aligns with experimental findings. This enhanced performance can be attributed to the effective adsorption and activation of NO_3_^−^ through the charge “acceptance‐donation” mechanism, as well as the moderate binding facilitated by the occupation of the d‐p antibonding orbital. Furthermore, the study revealed a strong correlation between eNO_3_RR activities and the intrinsic properties of TM centers and their local environments. By using the established activity descriptor, the researchers efficiently screened several other graphene‐based single‐atom catalysts (SACs) that exhibited excellent eNO_3_RR performance. The catalyst consisted of atomically dispersed Cu sites anchored on a dual‐mesoporous N‐doped carbon framework was reported by Xu.,^[^ ^165^ ^]^ which achieved an NH_3_ yield rate of 13.8 mol g_cat._^−1^ h^−1^ and a NO_3_^−^‐to‐NH_3_ FE of 95.5%. During a continuous 120‐h eNO_3_RR test in simulated NH_3_ synthesis conditions with a large current density of about 200 mA cm^−2^ and an amplified volume of NO_3_^−^ solution (9 times), the Cu−N−C catalyst demonstrated stability. DFT calculations indicated that the atomically dispersed Cu_1_−N_4_ sites contributed to reducing the energy barrier of the RDS in eNO_3_RR. Apart from Cu, single‐atom Fe and Ru also exhibit catalytic activity. Wu et al.^[^ ^136^ ^]^ reported selective and active NO_3_^−^‐to‐ NH_3_ reduction on a single‐atom Fe catalyst, achieving a maximum FE of ≈75% and a maximum yield rate of ≈20 000 µg h^−1^ mg_cat._^−1^ (0.46 mmol h^−1^ cm^−2^). The single‐atom Fe catalyst effectively inhibits the N*─*N coupling step necessary for N_2_ due to the absence of adjacent metal sites, thereby enhancing the selectivity toward NH_3_ production. DFT calculations uncover the reaction mechanisms and identify the RDS for eNO_3_RR on Fe sites with atomic dispersion. Yao et al.^[^ ^134^ ^]^ propose an intrinsic oxide anchoring method to securely attach ligand‐free isolated Ru atoms onto the amorphous layer of a monolithic Ti support through the regulation of electronic metal‐support interactions. The resultant single atom (SA) electrode composed of Ru displayed exceptional electrochemical chlorine evolution activity, exhibiting three orders of magnitude higher mass activity compared to that of a commercially available dimensionally stable anode. Additionally, it selectively converted NO_3_^−^ to NH_3_ at an NH_3_ yield rate of 22.2 mol g^−1^ h^−1^. Moreover, the SA monolithic electrode composed of Ru can be scaled up from dimensions of 2 × 2 cm to at least 25 × 15 cm, showcasing significant potential for various industrial electrocatalytic applications (**Figure** **13**).

Adjusting the type of nitrogen can also fine‐tune the coordination environment around SAs, ultimately enhancing reaction activity. For instance, Liu et al.^[^ ^132^ ^]^ demonstrated a pyridinic‐N‐rich Cu single‐atom catalyst (PR‐CuNC) derived from semi‐interpenetrating polypyrrole‐polyethyleneimine hydrogels for the eNO_3_RR. In contrast to the catalyst with insufficient pyridinic nitrogen, PR‐CuNC demonstrates a maximum NH_3_ FE of 94.61% and a yield rate of 130.71 mg mg_Cu_^−1^ h^−1^ (3.74 mg h^−1^ cm^−2^). DFT calculations reveal that different coordination types of nitrogen significantly affect the electronic structures of CuN_4_ sites, leading to enhanced intrinsic activity. Furthermore, utilizing non‐nitrogen atoms (such as O and P atoms) to break the symmetry of the coordination environment can also lower the energy barrier of certain steps in eNO_3_RR, thereby enhancing performance. For example, Cheng et al.^[^ ^166^ ^]^ designed Cu SACs with broken symmetry of coordination by substituting the local coordinating atoms from 4N to 2N + 2O. This breaks the coordination symmetry and leads to a more polar active site, thereby increasing the accumulation of NO_3_^−^ near the surface of the electrocatalyst. Additionally, the *cis*‐coordination leads to the splitting of Cu 3*d* orbitals, which generates a π‐complex of the intermediate *ONH with an orbital‐symmetry match. This, in turn, reduces the energy barrier compared to the σ‐complex generated using other catalysts. The symmetry‐broken Cu‐*cis*‐N_2_O_2_ SAC strike a good balance between catalytic activity and long‐term stability with an average NH_3_ yield rate of 27.84 mg h^−1^ cm^−2^, which is suitable for industrial‐level current densities of 366 mA cm^−2^. These results show that coordination symmetry plays a crucial role in SACs. Another example is that Xu et al.^[^ ^133^ ^]^ developed an SA Fe catalyst coordinated with N and P on a hollow carbon polyhedron (Fe‐N/P‐C) for eNO_3_RR. The effect on breaking local charge symmetry by P atoms of singe‐Fe‐atom catalyst facilitates the adsorption of NO_3_^−^ and enrichment of key reaction intermediates during eNO_3_RR. Consequently, the Fe‐N/P‐C catalyst achieves 90.3% NH_3_ FE with a yield rate of 17 980 µg h^−1^ mg_cat._^−1^. Various operando Synchrotron‐radiation FTIR spectroscopy measurements reveal the key intermediates in the reaction pathway under different applied potentials and reaction durations. Additionally, DFT calculations show that the optimized free energy of eNO_3_RR intermediates is the result of the asymmetric atomic interface configuration, achieving optimal electron density distribution. Li et al.^[^ ^167^ ^]^ developed a Co‐SAC with modified phosphorus and high atom efficiency using a defect‐rich carbon basal plane anchor. Modification with phosphorus improved the local environment of the Co atom through asymmetric charge distribution and electron redistribution. The unique tetracoordinated structure of Co‐SAC demonstrated high NH_3_ production efficiency and good cycle stability, making it a promising option for treating wastewater. Introducing metal single‐atom alterations to the metal coordination environment and utilizing synergistic effects to enhance catalytic activity represents an effective strategy for catalytic enhancement. A Pd metallene alloyed with SA Bi, as reported by Chen et al.,^[^ ^111^ ^]^ demonstrates an NH_3_‐FE approaching 100% and NH_3_ yield rate of 33.8 mg h^−1^ cm^−2^ at −0.6 V versus reversible hydrogen electrode (RHE). The results of DFT calculations and operando spectroscopic techniques reveal that SA Bi forms electronic coupling with neighboring Pd atoms, synergistically activating NO_3_^−^ and destabilizing *NO on Bi_1_Pd. This results in a lowered energy barrier for the RDS (*NO→ *NOH) and enhanced protonation energetics in the NO_3_^−^‐to‐NH_3_ conversion pathway. Liu et al.^[^ ^135^ ^]^ designed an electrocatalyst for eNO_3_RR comprising rhodium SAs dispersed onto copper nanowires, resulting in a partial current density of 162 mA cm^−2^ for NH_3_ production and an FE of 93% at −0.2 V versus RHE. The maximum NH_3_ yield rate reached a record value of 1.27 mmol h^−1^ cm^−2^. Detailed investigations using EPR, in situ infrared spectroscopy, DEMS and DFT modeling suggest that the high activity stems from the synergistic catalytic cooperation between rhodium and copper sites, whereby adsorbed hydrogen on the rhodium site transfers to vicinal *NO intermediate species adsorbed on copper, promoting hydrogenation and NH_3_ formation. Apart from utilizing SACs, dual‐atom catalysts can also alter the coordination environment. Shu et al.^[^ ^168^ ^]^ systematically investigates the performance of dual‐atom catalysts (denoted as M_1_M_2_@g‐CN) supported on graphitic carbon nitride (g‐CN) for the eNO_3_RR process by means of spin‐polarized first‐principles calculations. The heterogeneity of dual‐metal sites creates a synergistic effect, which can modulate the activity and selectivity for eNO_3_RR. FeMo@g‐CN and CrMo@g‐CN exhibit superior performance with respect to the 21 examined candidates, presenting low limiting potentials of −0.34 and −0.39 V, respectively. The activities can be attributed to the synergistic coupling effect between the M_1_M_2_ dimer *d* orbitals and the antibonding orbital of NO_3_^−^. Dissociating the deposited FeMo and CrMo dimers into separated monomers is shown to be challenging, thus ensuring the kinetic stability of M_1_M_2_@g‐CN.

**Figure 14:**
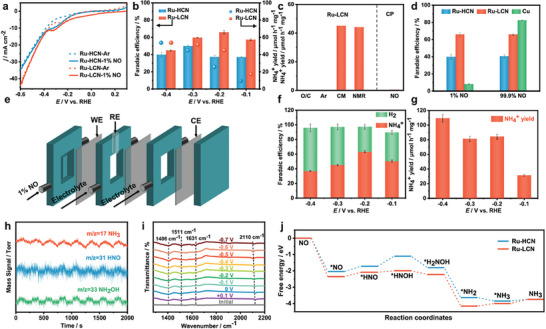
a) LSV curves of Ru‐HCN and Ru‐LCN in Ar‐ and 1% NO‐saturated electrolyte. b) FE and yield rate of NH_3_ at various potentials. c) eNORR performance of CP and Ru‐LCN under different conditions (O/C represents the removal of external potential), and ^1^H NMR spectra quantification. d) FE with 1% and 99.9% NO over various catalysts at −0.2 V versus RHE. e) Diagram of the flow electrolyzer for eNORR. f) FE and g) Yield rate with 1% NO on Ru‐LCN in a flow electrolyzer at each given potential. h) DEMS measurements of Ru‐LCN for eNORR at −0.2 V versus RHE. i) Potential‐dependent in situ FTIR spectra of Ru‐LCN for eNORR. j) Reaction Δ*G* diagram of eNORR over Ru‐HCN and Ru‐LCN on FCC (111) surfaces at 0 V versus RHE. Reproduced with permission.^[^ ^137^ ^]^ Copyright 2022, American Chemical Society.

**Figure 15:**
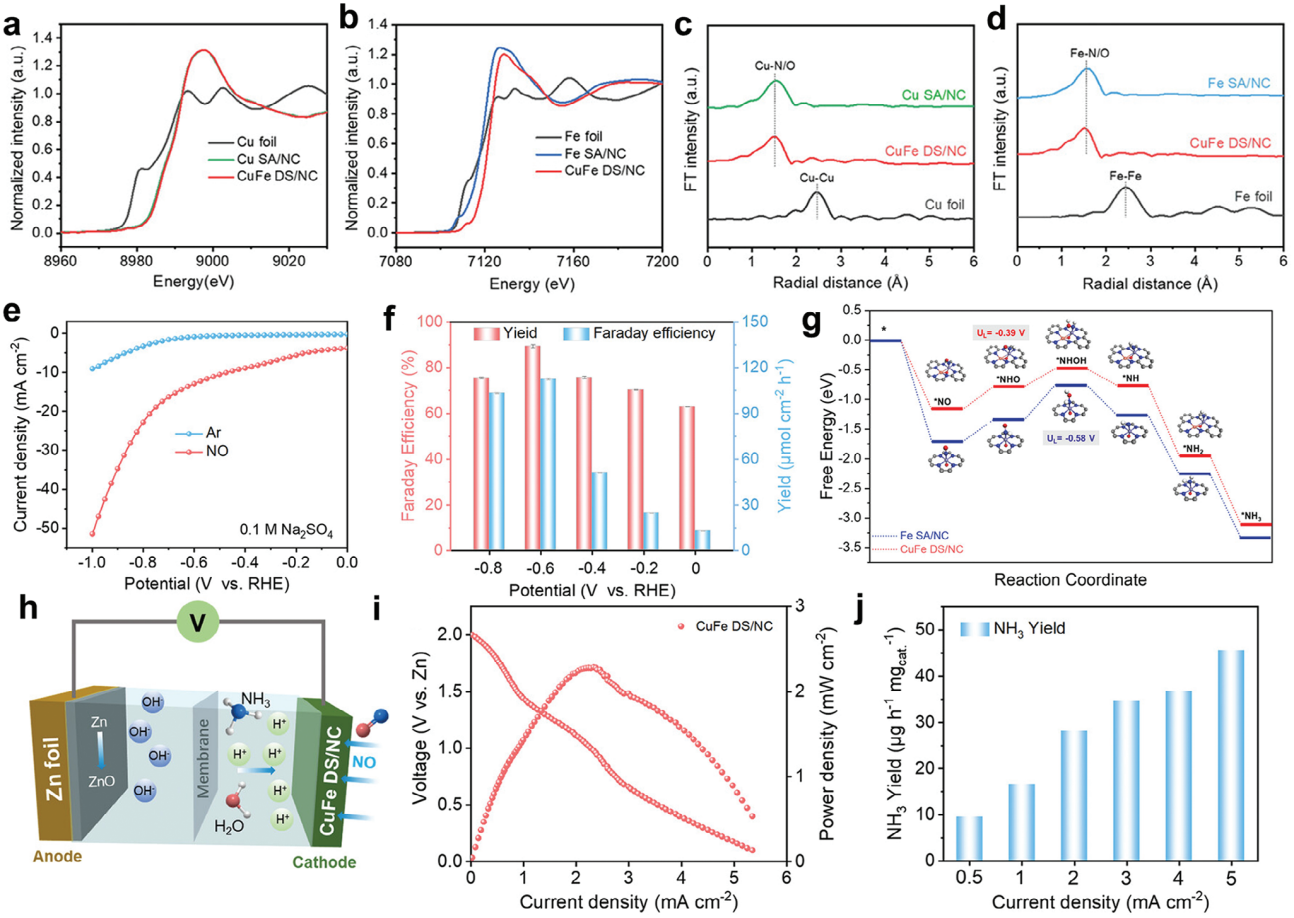
a) Normalized Cu K‐edge XANES and b) Fe K‐edge XANES spectra of different samples. Fourier transform EXAFS spectra of c) Cu‐based samples and d) Fe‐based samples. e) LSV curves of the CuFe DS/NC measured in Ar and NO‐saturated electrolytes. f) FEs and yield rates of NH_3_ measured on CuFe DS/NC at various potentials. g) The free energy diagram of eNORR on O ligand‐modified Fe SA/NC and CuFe DS/NC. h) Schematic diagram of Zn*─*NO battery. i) Power density and polarization curve of the CuFe DS/NC‐based Zn*─*NO battery. j) NH_3_ yield rates at different current densities. Reproduced with permission.^[^ ^138^ ^]^ Copyright 2023, Wiley‐VCH.

Yao et al.^[^ ^169^ ^]^ theoretically designed a type of catalyst that contains SA and is generated through a two‐step structural self‐regulation process. During the thermodynamic self‐regulation step, the presence of divacancies in graphene causes the spontaneous migration of SA from transition metal supports (*dv*‐*g*/TM; TM = *fcc* Co, *hcp* Co, Ni, Cu), resulting in a significantly high loading of SA. Meanwhile, this behavior generates TM vacancies in the supports. Furthermore, the coordination environment of the SA is altered because of the presence of adsorbate (i.e., NO* and the subsequent hydrogenated species during the eNORR) and vacancy migration, allowing the catalyst to bypass the limitations imposed by scaling relationships. As a result, the designed catalyst, *dv*‐*g*/Ni, exhibits efficient catalytic activity for the conversion of NO to NH_3_ at a low Δ*G* of the RDS. Combining machine learning (ML) with DFT calculations enables the screening of eNORR catalyst materials. A brand single‐cluster catalyst (SCC) of the TM*_n_* clusters (TM = Fe, Co, Ni and *n* = ≈1≈4) preadsorbed on 2D GaS nanosheet (TM*_n_* @GaS) were predicted by Yang et al.^[^ ^170^ ^]^ It is found that Co_4_@GaS can realize superior eNORR activity with a working potential of −0.06 V. As shown in **Figure** **14**, Ru nanosheets with low coordination numbers (Ru‐LCN) are prepared by Li et al via a plasma treatment of Ru nanosheets with high coordination numbers (Ru‐HCN).**^[^** ^137^ ^]^ And Ru‐LCN exhibits eNORR activity (NH_3_ yield rate, 45.02 µmol h^−1^ mg^−1^; FE, 65.96%) at −0.2 V versus RHE, which is higher than Ru‐HCN (NH_3_ yield rate, 25.57 *µ*mol h^−1^ mg^−1^; FE, 37.25%). The results of DEMS, electrochemical in situ FTIR spectroscopy, and DFT calculations reveal the possible reaction pathway and enhanced mechanism. Creating the low coordination number Ru active sites facilitates NO adsorption and reduces the energy barrier for RDS. Wang et al.^[^ ^138^ ^]^ reported an atomic Cu‐Fe dual‐site electrocatalyst (CuFe DS/NC) anchored on nitrogen‐doped carbon for eNORR (**Figure** **15**). The CuFe DS/NC catalyst exhibits a FE of 90% and a yield rate of 112.52 µmol cm^−2^ h^−1^. This performance surpasses the corresponding Cu single‐atom (NH_3_ yield rate, 61.08 µmol h^−1^ cm^−2^; FE, 63.83%), Fe single‐atom (NH_3_ yield rate, 73.90 µmol h^−1^ cm^−2^; FE, 74.70%). Furthermore, Zn*─*NO battery was assembled with CuFe DS/NC as the cathode, producing a power density of 2.30 mW cm^−2^. The DFT calculations show that bimetallic sites can enhance electrocatalytic eNORR by altering the RDS and accelerating the protonation process.

**Figure 16:**
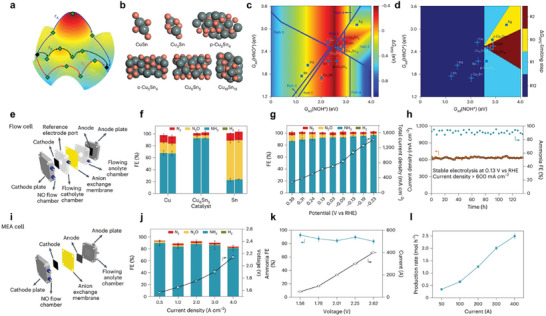
a) Schematic of the global energy optimization approach used to determine the eNORR activity from a complex reaction network (the redder the color shading, the higher the energy). b) Six Cu*─*Sn alloys with different crystal structures (the orange and gray balls represent Cu and Sn atoms, respectively). c) 2D (quasi) activity map with two independent descriptors, the adsorption free energies of HNO* and NOH*. The map is divided by blue lines, representing different pathways. The color bar shows the Δ*G*_RPD_‐limiting energies; the more negative the energy, the higher the activity. d) 2D map of the Δ*G*_RPD_‐limiting steps for the eNORR over different metals and alloys. The data are presented with indicative error bars to reflect a DFT uncertainty of ± 0.1 eV. e) Schematic illustration of the flow cell. f) Comparison of the FE of the Cu, Cu_6_Sn_5_, and Sn electrodes at nearly 0.03 (left) and −0.10 V (right) versus RHE in a flow cell. g) Potential‐dependent FE and total geometrical current density over Cu_6_Sn_5_ electrodes. h) Stability of the Cu_6_Sn_5_ electrode measured at 0.13 V versus RHE over 135 h. i) Schematic illustration of the MEA electrolyser. j) Current density‐dependent FE and voltage in an MEA electrolyser. k) FE for NH_3_ and current as a function of voltage in a scaled‐up MEA electrolyser. l) NH_3_ production rate as a function of current. Reproduced with permission^[^ ^172^ ^]^ Copyright 2023, Springer Nature.

A series of Ru‐doped Cu catalysts were synthesized via an in situ electroreduction of metal hydroxide precursors by Shi et al.^[^ ^171^ ^]^ The Ru_0.05_Cu_0.95_ exhibits better eNORR activity (NH_3_ yield rate, 17.68 µmol cm^−2^ h^−1^; FE, 64.9%), surpassing its Cu counterpart (NH_3_ yield rate, 5.73 µmol cm^−2^ h^−1^; FE, 33.0%). Ru doping‐induced downshift of the Cu *d*‐band center facilitates the rate‐limiting hydrogenation step and decreases the desorption energy of NH_3_, resulting in the improvement of FE and NH_3_ yield rate. Shao et al.^[^ ^172^ ^]^ designed Cu_6_Sn_5_ alloy that exhibits eNORR activity by tuning the electronic properties of Cu (**Figure** **16**). In a flow cell, the yield rate of NH_3_ reached 10 mmol cm^−2^ h^−1^, showcasing an FE exceeding 96% at a current density surpassing 1400 mA cm^−2^. Furthermore, it demonstrated stability at a current density exceeding 600 mA cm^−2^ with an NH_3_ FE of ≈90% over a period of 135 h. In a scaled‐up membrane electrode assembly electrolyser, the rate of NH_3_ production achieved ≈2.5 mol h^−1^, fueled by a current of 400 A at an operating voltage close to 2.6 V. The EAS performances of partial electrocatalysts enhanced by coordination environment modulation are listed in Table 5.

### 4.6 The Others

The aforementioned strategies focus on catalyst design to enhance activity. In the context of EAS in aqueous systems, electrolyte design also plays a pivotal role in improving reaction efficiency. For instance, Shen et al.^[^ ^173^ ^]^ integrated catalyst and electrolyte engineering to achieve high‐efficiency eN_2_RR using a selenium‐vacancy‐rich WSe_2−_ *_x_* catalyst in a water‐in‐salt electrolyte (WISE). It is revealed that WISE suppresses HER, enhances N_2_ affinity on the catalyst surface, and improves the π‐back‐donation capability of active sites, thereby boosting both activity and selectivity for eN_2_RR. This approach yielded a remarkable FE of 62.5% and an NH_3_ production rate of 181.3 µg h^−1^ mg^−1^ in 12 m LiClO_4_. Wang et al.^[^ ^174^ ^]^ demonstrated that a salting‐out effect induced in a highly concentrated electrolyte can mitigate the competing HER and facilitate efficient NH_3_ synthesis. The solute ions exhibit strong interactions with surrounding H_2_O molecules, forming hydration shells that limit their availability as proton sources and solvents. This mechanism not only suppresses HER but also ensures substantial nitrogen flux at the reaction interface through heterogeneous nucleation of precipitates, thereby enhancing both selectivity and activity. Notably, even when paired with a metal‐free electrocatalyst, this proof‐of‐concept system achieved a high FE of 71% ± 1.9%.

**Figure 17:**
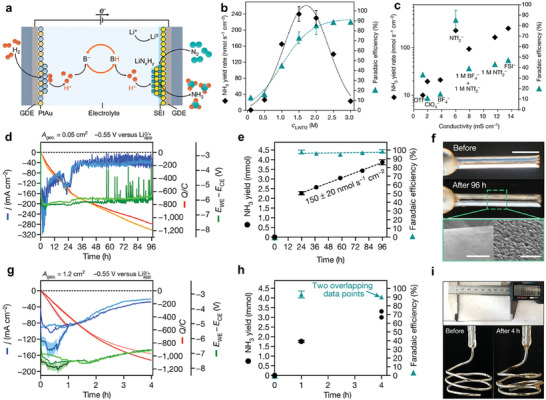
a) Schematic proton‐shuttling process for Li‐eN_2_RR in a continuous‐flow electrolyser. Reproduced with permission.^[^ ^175^ ^]^ Copyright 2024, Springer Nature. b) Comparison of the Li‐eN_2_RR FE (triangles) and NH_3_ yield rate (diamonds) (lines are guides to the eye) as a function of LiNTf_2_ concentration. c) Li‐eN_2_RR FE (triangles) and NH_3_ yield rate (diamonds) (note the log scale) plotted against conductivity for different electrolytes at 2 m, a mixed 1 m LiNTf_2_ + 1 m LiBF_4_ system, and 1 m LiNTf_2_. d) Two uninterrupted 96 h potentiostatic tests. e) The evolution of the NH_3_ yield (circles) and Li‐eN_2_RR FE (triangles) over time for uninterrupted (24 and 96 h) and interrupted (37.2, 58.4, 78.2, and 96 h) experiments. f) Photographs and SEM images of the electrode before and after an uninterrupted 96 h experiment. g) Reproducibility of 1 h chronoamperograms shown as the mean (solid lines) and s. d. (shading) of *n*  =  4 independent repeats, and two 4 h potentiostatic tests. h) The corresponding evolution of the NH_3_ yield (circles) and Li‐eN_2_RR FE (triangles) for the 1 and 4 h experiments. i) Photographs of the electrode before and after a 4 h experiment. Reproduced with permission.^[176]^ Copyright 2022, Springer Nature.

In nonaqueous systems for Li‐eN_2_RR, electrolyte regulation also significantly improves reaction efficiency. This method is unique among catalytic processes, as both N_2_ reduction and protonation depend on and occur within the SEI layer, which is critical for mediating catalysis. The process involves three key steps (**Figure** **17**a): 1) electrochemical deposition of metallic Li, 2) direct reaction of Li with N_2_ to form Li_3_N, and 3) spontaneous decomposition of Li_3_N into NH_3_ and Li^+^ in the presence of protons. By bypassing the HER competition inherent to aqueous eN_2_RR, this method achieves an initial current efficiency of up to 72%.^[^ ^175^ ^]^ In Li‐eN_2_RR, the SEI layer plays a dual role: stabilizing the Li metal surface and participating in the catalytic reaction. Its formation and stability are essential for uniform Li deposition, N_2_ adsorption and activation, and overall reaction kinetics. Compared to Li‐ion batteries, the SEI in Li‐eN_2_RR has distinct functions and characteristics. While in Li‐ion batteries, the SEI primarily stabilizes the Li metal surface and prevents electrolyte decomposition, in Li‐eN_2_RR, it must also optimize N_2_ reduction kinetics and selectivity to maximize NH_3_ yield rate and FE. The formation mechanisms, electrochemical performance, and interface stability of the SEI differ significantly between these two systems. Therefore, the role of the electrolyte and a high‐efficiency, robust process was investigated that is enabled by compact ionic layering in the electrode–electrolyte interface region.^[^ ^176^ ^]^ The interface is induced by a high‐concentration imide‐based lithium‐salt electrolyte, providing maintained NH_3_ yield rates of 150 ± 20 nmol s^−1^ cm^−2^ and a current‐to‐ NH_3_ efficiency that is close to 100%. The ionic assembly formed at the electrode surface suppresses the electrolyte decomposition and supports stable Li‐eN_2_RR (Figure 17).

**Figure 18:**
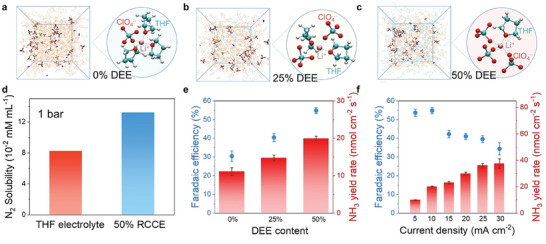
The final snapshots of the simulation boxes with the primary solvation structures (right) of electrolytes with a) 0%, b) 25%, and c) 50% DEE content. d) The N_2_ solubility of THF electrolyte and 50% RCCE under 1 bar. e) The calculated FEs and NH_3_ yield rates with the increasing DEE content. f) Li‐eN_2_RR performance of 50% RCCE under various current densities. Reproduced with permission.^[^ ^177^ ^]^ Copyright 2023, Wiley‐VCH.

**Figure 19:**
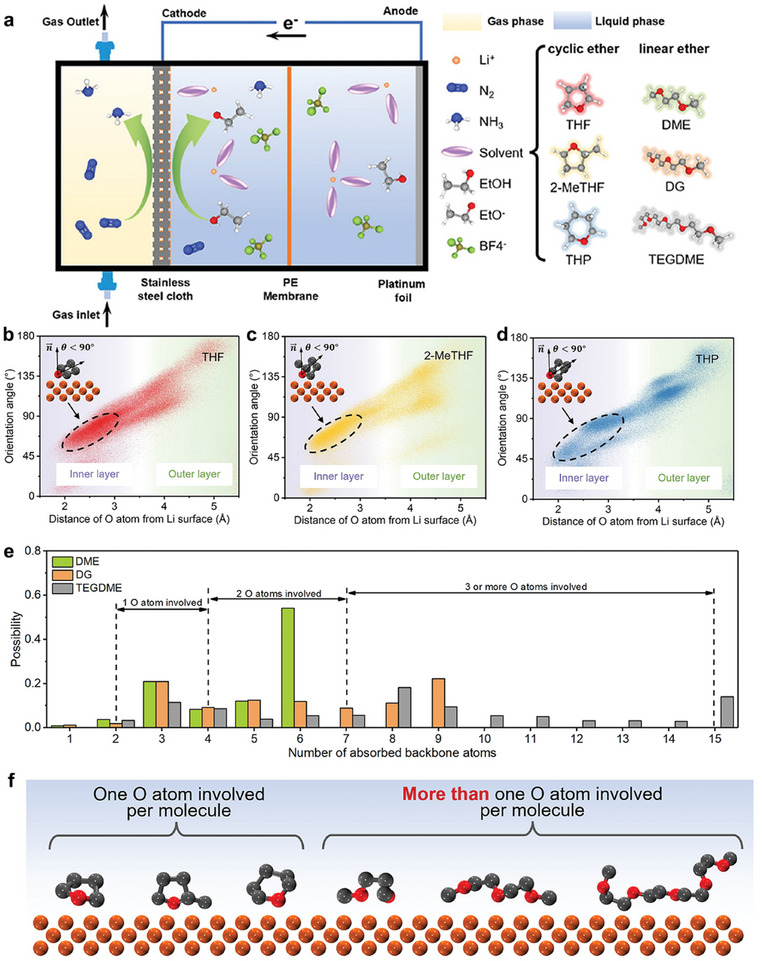
a) Illustration of the gas diffusion cell. b–d) Orientation angle of cyclic ethers above the lithium plate. e) Probability distribution of linear ethers with different numbers of absorbed backbone atoms on the lithium plate. f) Schematic diagram of typical configurations of varied cyclic and linear ethers on the lithium plate. Reproduced with permission.^[^ ^180^ ^]^ Copyright 2023, American Chemical Society.

Building upon the lithium battery electrolyte achievement, the SEI problem in lithium electrochemistry poses a significant obstacle to the further enhancement of Li‐eN_2_RR performance. Li et al.**^[^** ^177^ ^]^ apply the ring‐chain solvents coupling law to the Li‐eN_2_RR system, thereby optimizing the interface and achieving a nearly twofold increase in FE, reaching 54.78% ± 1.60% (**Figure** **18**a–f). Systematic theoretical simulations and experimental analysis collectively reveal that the anion‐rich Li^+^ solvation structure, resulting from the coupling of ring tetrahydrofuran with chain ether, effectively mitigates excessive passivation of electrolyte decomposition at the reaction interface. Consequently, this promotes the mass transfer of active species and enhances the kinetics of nitrogen fixation. Au‐coated carbon fibrous paper (Au/CP) was selected as the catalyst model to investigate the Li‐eN_2_RR activities on different catalytic surfaces. In situ XRD analysis confirmed the conversion of Li intermediates during Li‐eN_2_RR. Au significantly enhanced the kinetics of electron transfer to catalyze the formation of metallic Li. The FE of Li‐eN_2_RR on Au/CP was found to be 34.0% ± 4.5% with an NH_3_ yield rate reaching as high as 47.2 ± 1.2 µg h^−1^ cm^−2^.^[^ ^178^ ^]^ Li et al.^[^ ^179^ ^]^ focused on investigating the dynamic changes of the SEI under various experimental conditions and elucidating the influence of the SEI layer on enhancing the performance of the Li‐eN_2_RR process through the modulation of ionic conductivity. Among several electrolytes, a fluorine‐based electrolyte exhibits superior performance. By utilizing a porous Cu electrode, an FE of 95% ± 3% with a current density of −0.1 A cm_geo_^−2^ under 20 bar N_2_ was achieved. Furthermore, a FE of 71% ± 3% can be attained with a current density of −1.0 A cm_geo_^−2^, resulting in an NH_3_ production rate of 2.5 ± 0.1 µmol s^−1^ cm_geo_^−2^. This can be attributed to the presence of a homogeneous SEI layer enriched with LiF, which promotes uniform Li that facilitates even Li deposition and suppresses the uncontrolled electrolyte degradation. A significant performance improvement in Li‐eN_2_RR has been achieved in recent years by the exploration of favorable Li salt and proton donor for the electrolyte recipe, but the solvent study is still in its infancy. Cai et al.**^[^** ^180^ ^]^ systematically investigated ether‐based solvents for Li‐eN_2_RR, evaluating solvent candidates based on their conductivity, parasitic reactions, product distribution, and FE (**Figure** **19**). The solvent molecules induce the formation of SEI with different morphologies and compositions. Notably, dimethoxyethane demonstrates the least potential loss among the investigated systems, whereas tetrahydrofuran achieves exceptional FE of 58.5% ± 6.1% at ambient pressure.

In addition to the electrolyte, the reactor design is also pivotal. Xu et al.^[^ ^181^ ^]^ proposed a stepwise strategy to construct a bipolar membrane (BM) with stable C*─*C covalent interlocking (CIBM), which significantly enhances physical binding strength and ionic transport rates. This covalent design enables exceptional water dissociation (WD) performance under high current density (1.17 V at 1000 mA cm^−2^) and unprecedented stability (1100 h). The introduction of the covalent interface also facilitated continuous BM NH_3_ electrosynthesis with high efficiency, low energy consumption, and state‐of‐the‐art yield rate (70.9 mg cm^−2^ h^−1^) from 2000 ppm NO_3_^−^, offering innovative design principles for emerging ampere‐level BM electrochemical devices. Ge et al.^[^ ^182^ ^]^ presented a continuous flow plasma‐electrochemical reactor system for the direct conversion of atmospheric N_2_ into NH_3_. In this system, N_2_ molecules are initially converted into NO*_x_* species in the plasma reactor, which are subsequently fed into the electrochemical reactor. To selectively convert NO*_x_* species into NH_3_, a graph theory approach combined with first‐principles calculations was employed to enumerate all possible pathways from N_2_ to NH_3_, identifying critical intermediates (NH_2_* and NO*). Bimetallic catalysts were then designed to optimize the adsorption and conversion of these key intermediates. Using an optimized CuPd foam catalyst, the system achieved an NH_3_ production rate of 81.2 mg h^−1^ cm^−2^ with stability over 1000 h at an applied current of 2 A.

Besides, the design of cathode/reference electrodes can also be a strategy to improve Li‐eN_2_RR performance. To enhance the current density, Li et al.^[^ ^183^ ^]^ synthesized high‐surface‐area Cu electrodes using hydrogen bubbling templating (HBT). The HBTCu electrode, which exhibits a refined micro‐nano structure. In a home‐built autoclave under 20 bar of N_2_, a current density of ≈100 mA cm_geo_^−2^ was successfully attained, resulting in an NH_3_ FE of 13.3%. Increasing the concentration of LiClO_4_ demonstrated enhanced system stability, which is attributed to a change of Li deposition and/or solid electrolyte interphase. Furthermore, Lazouski et al.^[^ ^184^ ^]^ reported a method to address transport limitations in tetrahydrofuran (THF) by employing a stainless‐steel cloth (SSC)‐based support for NH_3_ synthesis combined with hydrogen oxidation. They achieved a gas diffusion electrode by controlling material wetting and electrolyte penetration into supports when utilizing nonaqueous electrolytes. Control of electrolyte penetration was achieved by maintaining a pressure gradient across the cloth. This method efficiently oxidized hydrogen on platinum‐coated SSCs at current densities of up to 25 mA cm^−2^ in both THF‐ and propylene carbonate‐based electrolytes. Additionally, the Li‐eN_2_RR can be achieved by employing an SSC as a substrate onto which lithium metal is plated in situ. An NH_3_ partial current density of 8.8 ± 1.4 mA cm^−2^ and high FE (35 ± 6% at rate‐optimized conditions, 47.5% ± 4% at FE ‐optimized conditions) were obtained. Cai et al.^[^ ^185^ ^]^ proposed a feasible configuration of a membrane electrode assembly (MEA), which shows promise in overcoming the problems of poor gas transfer, dependence on organic solvent and significant voltage loss. The MEA consisted of stainless‐steel cloth with deposited lithium as the cathode, lithium‐doped polyethylene oxide as the polymer electrolyte, and carbon paper loaded with a Pt/C catalyst as the anode. At a cell voltage of 3.6 V, an average NH_3_ production rate of 2.41 ± 0.14 µmol h^−1^ cm^−2^_geo_ and a FE of 8.9 ± 1.7% were achieved. The absence of ethanol resulted in lower voltage loss (≈0.25 V @ 5 mA cm^−2^_geo_). This study proposes a new approach to Li‐eN_2_RR, providing advantages such as efficient gas transfer, reduced solvent consumption, and a compact configuration. Protonation is one of the key steps in Li‐eN_2_RR, making the design of proton shuttle substances particularly important. To resolve the limitation of the required sacrificial source of protons in Li‐eN_2_RR, A phosphonium‐based ionic liquid was introduced to act as a reliable proton shuttle. This salt also provides additional ionic conductivity, enabling high NH_3_ production rates of 53 ± 1 nmol s^−1^ cm^−2^ at 69 ± 1% FE in 20 h experiments under 0.5‐bar hydrogen and 19.5‐bar nitrogen.^[^ ^186^ ^]^

## 5 Perspective and Outlook

In this review, the progress of three routes for EAS was summarized and the strategies to improve the NH_3_ yield rates and FE were also discussed. Although many electrocatalysts for EAS have been designed, the yield rates and FEs also have been improved, there are still many challenges that remains to be solved, details are listed as follows:

For eN_2_RR, first, it is necessary to establish strict control experiments for reliable eN_2_RR measurements. In recent years, eN_2_RR has developed rapidly, and various electrocatalysts and electrocatalytic systems have been continuously developed and reported. The records of yield rate and FE have been constantly broken and updated. However, due to the extremely low NH_3_ yield rates and FEs in the current eN_2_RR, it is highly susceptible to contaminations such as NH_3_ and nitrogen‐containing species that are prevalent in the external environment. Therefore, it is important to conduct rigorous controlled experiments, especially using ^15^N_2_ isotope measurements to track the N source of NH_3_ production. In addition, researchers are recommended to provide all experimental details when publishing their work to ensure reproducibility and to provide a solid foundation for the development of this emerging field. Second, a suitable eN_2_RR benchmark catalyst should be selected as a criterion for evaluating catalysts. It is well known that commercially available electrocatalysts, such as Pt/C, RuO_2_, and IrO_2_ are widely used as benchmark catalysts for HER, ORR, and OER. However, there is still no benchmark catalyst in eN_2_RR. It is difficult for researchers to assess whether current systems are scientifically reliable, leading to a large number of cases where results are unreliable and difficult to reproduce. In order to obtain meaningful results, there is an urgent need to benchmark eN_2_RR catalysts so that researchers can check whether the observed experimental performance matches the literature data. Third, using the latest in situ characterization techniques in eN_2_RR. Currently, there are relatively limited reports on the experimental detection of the reaction intermediate species. From this perspective, high‐level in situ or operational characterization techniques will reveal the in‐depth relationship between the reactivity and important limiting parameters, as well as the dynamic changes occurring on the catalyst surface during the electrochemical reaction.^[^ ^187^ ^]^ For example, quasi in situ XPS measurements can be used to identify reaction intermediates and their interactions with the catalyst during the eN_2_RR process. With the development of advanced in situ technologies, the reaction mechanism can be explored in depth, providing further guidance for the rational optimization of catalysts. Finally, eN_2_RR is currently limited due to the difficulty of N_2_ activation. Notably, multistep N_2_ fixation with NH_3_ immobilization that effectively solves one problem at each step has recently become the latest frontier in the field of eNRR. For example, two‐step N_2_ to NH_3_ fixation can be rationalized by linking plasma‐driven N_2_ oxidation activation of N_2_ in tandem with an electrochemical NO*_x_* reduction process, resulting in highly selective NH_3_ generation.^[^ ^188^ ^]^ These findings provide a reasonable paradigm for catalytic N_2_ fixation and encourage the development of additional alternative processes for NH_3_ production. For Li‐eN_2_RR, the development of in situ/operando techniques (XRD, XPS, XAS, and FTIR) will help to reveal the mechanism of Li‐eN_2_RR and identify the intermediate substances. For example, Gao et al.^[^ ^178^ ^]^ observed the presence of Li_3_N signals on carbonized carbon fiber paper by in situ XRD characterization during Li‐eN_2_RR. In addition, it is essential to establish standardized testing protocols for evaluating the performance of Li‐eN_2_RR across different reactor designs. This will not only facilitate more direct comparisons of NH_3_ yield rate and FEs but also promote the development and optimization of reactor designs tailored to specific conditions. By harmonizing testing methodologies, researchers can more effectively build upon the work of each other, leading to faster advancements in this promising area of sustainable NH_3_ synthesis.

For eNORR, NO is a highly reactive chemical molecule. This chemical property determines its relatively easy formation of coupling products when coelectrolyzed with some organic and inorganic compounds. Hence, apart from C*─*N bonds, developing more compounds capable of forming new chemical bonds with NO may expand the scope of potential applications and catalytic transformations in various fields. To increase the diversity and value of coupling products, it is necessary to design corresponding catalytic active sites for different reaction systems, achieving synergistic adsorption and efficient activation of multiple reactant molecules. Additionally, elucidating and understanding the migration patterns of reaction intermediates are crucial for achieving high‐selectivity single‐product couplings. Therefore, in situ utilization of spectroscopic techniques to monitor the dynamic evolution of adsorbates and active sites on catalyst surfaces under operating conditions is necessary. Isotopic tracing methods and theoretical calculations aid in elucidating the mechanisms of electrocatalytic NO conversion. Besides, research on eNORR is still in its early stages. Most studies are conducted using simple solution three‐electrode systems, and the yield of target products is far from satisfactory. Therefore, to convert NO exhaust on a larger scale, the use of electrochemical devices such as gas diffusion electrodes, flow batteries, and Zn–NO batteries can achieve the goals of waste gas treatment, resource recovery, and sustainable development. This promotes innovation and application of electrochemical technology, holding significant environmental and economic implications.

For eNO_3_RR, on the one hand, there is a lack of standardized performance evaluation of eNO_3_RR, including the concentration of supporting electrolyte and NO_3_^−^ concentration, which hinders the comparison and study of the performance of the electrocatalysts. In particular, the NO_3_^−^ concentrations have been inconsistent in previous studies. Some studies have used low concentrations of NO_3_^−^ (<100 ppm), while others have used high concentrations (0.1 or 1 mol L^−1^). The choice of NO_3_^−^ concentration should be tailored to the different applications. Low NO_3_^−^ concentrations are preferred for wastewater treatment, and high NO_3_^−^ concentrations are preferred for NH_3_ synthesis and hydrogen storage. On the other hand, potential‐induced self‐reconfiguration is a common phenomenon in eNO_3_RR, especially for Cu‐containing catalysts, from the viewpoint of catalyst research. Once self‐reconstruction occurs, the composition and structure of the electrocatalysts change significantly, and the prepared materials should be regarded as “precatalysts,” and the generated new components should be the real active sites in eNO_3_RR. Neglecting self‐reconstruction leads to misidentification of the true active site, which ultimately misleads the study of the catalytic mechanism. Therefore, researchers should pay special attention to the changes in the surface composition and structure of the electrocatalyst after the catalytic process. This poses a challenge to enable more accurate in situ characterization for identifying the active site and the reaction pathway for eNO_3_RR. Lack of experimental evidence to assist DFT calculations reduces the reliability and accuracy of reaction mechanism elaboration. Although DEMS has been developed and used for the identification of intermediates, it is a technique for the detection of volatiles and the results do not directly reflect the structure of the adsorbed substances on the electrode surface. In situ FTIR or Raman spectroscopy combined with the spectral isotope effect can provide a viable strategy for determining the structure of adsorbed substances. Utilizing the isotope effects of N, O, and H, bands in in situ FTIR or Raman spectra can be identified. For example, when ^14^NO_3_^−^ is replaced by ^15^NO_3_^−^ as the nitrogen source, the wavenumbers of the bands associated with nitrogen in the in situ FTIR or Raman spectrum are shifted so that these bands can be assigned to the nitrogen adsorbent.

To further enhance the efficiency of EAS, beyond rigorous and in‐depth research on the mechanisms of various EAS reactions, it is essential to address several critical issues. Among these, the design of electrolytic cells for EAS is a primary focus. The cells provide a stable environment necessary for the reactions and their structure directly influences the arrangement of anode and cathode chambers and electrode placement, necessitating thoughtful design for practical applications. Recent advancements have explored five main types of electrolytic cells for EAS: single‐chamber cells, H‐type cells, back‐to‐back cells, polymer electrolyte membrane (PEM)‐type cells, and electrochemical flow cells. Specifically regarding eN_2_RR or eNORR, PEM cells provide better proton supply than back‐to‐back cells. However, proton transfer in PEM cells is limited due to the absence of electrolytes in the cathode chamber. Drawing inspiration from H‐type electrolytic cells, a promising approach is to modify PEM cells by incorporating a gas diffusion chamber and adjusting the electrolyte volume of the cathode chamber. This separates the gas and liquid cathode chambers, theoretically allowing efficient N_2_/NO and proton supply while preserving the advantages of gas–solid interface adsorption and activation. It is crucial to consider how the electrode‐membrane distance affects proton transport and input voltage in such designs. Additionally, the requirements for hydrophilicity vary on different sides of the gas‐diffusion electrode (GDE). The GDE consists of a gas diffusion layer (GDL), a microporous layer (MPL), and a catalyst layer (CL). The side in contact with the gas must exhibit high hydrophobicity to enable effective diffusion of N_2_/NO and to maintain the separation between the gas and liquid cathode chambers. In contrast, the side of the GDE that interfaces with the electrolyte requires a certain degree of hydrophilicity to enhance proton transport efficiency. This design enhances the concentration of N_2_/NO on the catalyst surface, reduces HER, facilitates product release, and accelerates the electrocatalytic fixation of N_2_/NO.^[^ ^189^ ^]^ However, the use of nonaqueous electrolytes can cause them to infiltrate carbon fibers, leading to GDL flooding and impeding gas diffusion. To address this challenge, stainless steel mesh is commonly used instead of carbon fiber structures, and controlled pressure is applied in the gas chamber to maintain the three‐phase interface, thereby improving the efficiency of the EAS. Additionally, increasing the pressure or optimizing the temperature can substantially enhance the adsorption and activation of N_2_/NO at the gas–solid interface.

On the other hand, the efficient screening of highly active catalysts through theoretical calculations can significantly enhance the efficiency of EAS. Presently, catalyst development is significantly constrained by the reliance on chemical intuition and empirical data, often necessitating extensive trial‐and‐error experiments across a vast materials space. This approach poses a significant barrier to the swift identification of superior catalysts. Addressing this challenge, descriptors rooted in the adsorption free energy of intermediates, derived from DFT calculations, have been extensively used to predict EAS activity. Nonetheless, such methods often apply only to specific surface configurations, and scaling relationships frequently impose limitations on catalyst performance, leading to discrepancies between theoretical forecasts and experimental results. This highlights the urgency of overcoming scaling relationships to unlock the potential of high‐performance electrocatalysts. Moreover, machine learning (ML) holds substantial promise in revolutionizing the collection and analysis of high‐dimensional data, uncovering latent statistical trends that can guide the identification of efficient catalysts from a vast array of candidate materials. Such breakthroughs have the potential to enhance catalyst design, experimental synthesis, and related domains. However, the establishment of a robust “big data” infrastructure supported by high‐performance computing and the comprehensive analysis of experimental data remain both promising and complex engineering tasks. Additionally, the creation of universally applicable ML models tailored to diverse EAS systems is a critical requirement for advancing the field.

Beyond the realm of catalyst optimization, the advancement of EAS technology hinges on the resolution of challenges related to NH_3_ separation within electrolytes. Innovations in electrolyte formulations or the development of tailored electrochemical cell architectures could enable the direct separation of NH_3_, streamlining downstream processing. Integrating membrane separation technologies with electrochemical methods offers another viable strategy for selectively extracting and concentrating NH_3_, thereby improving the economic feasibility and sustainability of the process. Future endeavors should focus on advancing and integrating these separation technologies to accelerate the practical deployment of EAS.

In summary, the three types of EAS reactions utilize different forms of nitrogen as substrates. The eN_2_RR begins with N_2_, while the eNORR and eNO_3_RR involve intermediate forms in the nitrogen cycle as substrates, achieving NH_3_ synthesis through multielectron transfer reactions. Each method presents distinct advantages and disadvantages. eN_2_RR benefits from a wide range of raw material sources and high product selectivity; however, the activation of the N≡N triple bond and the low solubility of N_2_ in solution lead to lower yield rates and FEs, posing challenges in meeting practical demands. When the nitrogen source is substituted with NO, eNORR becomes an alternative approach for NH_3_ synthesis. NO can be derived from automobile and industrial emissions, offering the dual benefits of environmental remediation and NH_3_ production. However, this reaction pathway is complex, involving multiple intermediate products, which complicates control and optimization for high‐performance NH_3_ synthesis. In the case of eNO_3_RR to NH_3_, it shares similar advantages with eNORR. NO_3_^−^ can be sourced from contaminated water, and its reduction to NH_3_ contributes to environmental cleanup as well as the production of high‐value products. Additionally, NO_3_^−^ exhibits higher solubility in water compared to N_2_ and NO, facilitating its activation. However, the eNO_3_RR process generates a wider variety of by‐products (e.g., NO_2_^−^, N_2_, NH_2_OH), necessitating further separation to isolate the main product. Overall, while electrochemical reduction reactions show great promise for sustainability and targeted processing, they encounter significant challenges related to efficiency, catalyst development, and energy consumption. Advances in technology and catalysts are essential for overcoming these limitations and facilitating the widespread application of these processes.

**Table 1:** Comparison of EAS performance of recent electrocatalysts enhanced by vacancy engineering (NA represent not available).

Electrocatalysts	NH_3_ yield rate^a)^	FE [%]^b)^	Increase fold (NH_3_ yield rate/FE)^c)^	Method	Refs.
Fe_3_C/Fe_3_O_4_@C‐950	25.7 µg h^−1^ mg^−1^	22.5	3.0/3.8	eN_2_RR	[86]
MoO_2_/C700	173.7 µg h^−1^ mg^−1^	27.6	20.4/10.2	eN_2_RR	[87]
MoO_3−_ *_x_*/MXene	95.8 µg h^−1^ mg^−1^	22.3	7.5/3.2	eN_2_RR	[88]
PCN‐NV4	8.09 µg h‐1 mg^−1^	11.59	10.9/NA	eN_2_RR	[89]
NV‐W_2_N_3_	11.66 ± 0.98 µg h^−1^ mg^−1^	11.67 ± 0.93	2.5/1.2	eN_2_RR	[90]
P‐NV‐C_3_N_4_	28.67 µg h^−1^mg^−1^	22.15	5.4/NA	eN_2_RR	[91]
La_0.9_FeO_3−δ_	22.1 µg h^−1^mg^−1^	25.6	2.2/1.6	eN_2_RR	[146]
B‐MnO_2_/CC	54.2 µg h^−1^mg^−1^	16.8	6.4/NA	eN_2_RR	[147]
H‐CeO_2_	25.64 µg h^−1^mg^−1^	6.3	2.3/NA	eN_2_RR	[149]
CoFe_2_O_4_ nanocube	30.97 µg h^−1^mg^−1^	11.65	2.4/2.0	eN_2_RR	[150]
BiVO_4_	8.60 µg h^−1^mg^−1^	10.04	2.6/2.6	eN_2_RR	[151]
TiO_2−_ *_x_*	0.045 mmol h^−1^ mg^−1^	85	1.9/1.3	eNO_3_RR	[49]
Fe_2_TiO_5_ nanofiber	0.73 mmol h^−1^ mg^−1^	87.6	1.2/1.1	eNO_3_RR	[117]
CoTiO_3−_ *_x_*	30.4 mg h^−1^ mg^−1^	92.6	1.6/1.5	eNO_3_RR	[129]
CoNi‐Vp‐1.0	0.0977 mmol h^−1^ cm^−2^	84.27	2.5/2.1	eNO_3_RR	[131]

^a)^ NH_3_ yield rate at the optimal potential;

^b)^ FE at the optimal potential;

^c)^ Fold increase in performance after vacancy engineering regulation.

**Table 2:** Comparison of EAS performance of recent electrocatalysts enhanced by crystal facet design (NA represent not available).

Electrocatalysts	NH_3_ yield rate^a)^	FE [%]^b)^	Increase fold (NH_3_ yield rate/FE)^c)^	Method	Refs.
Pd cubes	24.3 µg mg^−1^ h^−1^	36.6	5.3/21.5	eN_2_RR	[93]
Pt_3_Fe NWs/C	18.3 µg h^−1^ mg^−1^	12.3	4.1/3.4	eN_2_RR	[94]
Cu‐NBs‐100	650 mmol h^−1^ g^−1^	95	4.4/1.1	eNO_3_RR	[109]
Pd cuboctahedron/C	307 µg h^−1^ mg^−1^	35.1	10.5/3.8	eNO_3_RR	[156]
hcp‐Co	439.50 µmol cm^−2^ h^−1^	72.58	3.1/1.3	eNORR	[139]

^a)^ NH_3_ yield rate at the optimal potential;

^b)^ FE at the optimal potential;

^c)^ Fold increase in performance after crystal facet design.

**Table 3:** Comparison of EAS performance of recent electrocatalysts enhanced by hybridization engineering (NA represent not available).

Electrocatalysts	NH_3_ yield rate^a)^	FE [%]^b)^	Increase fold (NH_3_ yield rate/FE)^c)^	Method	Refs.
Co*_x_* Ni_3−_ *_x_* (HITP)_2_/BNSs‐P	128.26 µg h^−1^ mg^−1^	52.92	2.2/1.5	eN_2_RR	[95]
NiCoP/CoMoP/Co(Mo_3_Se_4_)_4_@C/NF	24.54 µg h^−1^ cm^−2^	23.15	13.3/22.7	eN_2_RR	[96]
Fe_1_S*_x_* @TiO_2_	18.3 µg h^−1^ mg^−1^	17.3	5.1/8.1	eN_2_RR	[102]
MoSe_2_/Ti_3_C_2_T*_x_*	56.96 µg h^−1^ mg^−1^	14.08	1.6/2.5	eN_2_RR	[106]
CrP/NPC	22.56 µg h^−1^ mg^−1^	16.37	1.5/NA	eN_2_RR	[107]
FeS_2_@TiO_2_/TP	330.3 µmol h^−1^ cm^−2^	97.0	2.5/1.7	eNO_3_RR	[123]
a‐B_2.6_C@TiO_2_/Ti	3678.6 µg h^−1^ cm^−2^	87.6	1.5/1.0	eNORR	[142]
CoB/Co@C	315.4 µmol h^−1^ cm^−2^	≈70	2.6/1.5	eNORR	[160]

^a)^ NH_3_ yield rate at the optimal potential;

^b)^ FE at the optimal potential;

^c)^ Fold increase in performance after hybridization engineering.

**Table 4:** Comparison of EAS performance of recent electrocatalysts enhanced by phase engineering (NA represent not available).

Electrocatalysts	NH_3_ yield rate^a)^	FE [%]^b)^	Increase fold (NH_3_ yield rate/FE)^c)^	Method	Refs.
Amorphous BiNi	17.5 µg h^−1^ mg^−1^	13.8	1.4/1.3	eN_2_RR	[97]
Amorphous IrTe	34.6 µg h^−1^ mg^−1^	11.2	2.3/3.9	eN_2_RR	[98]
B‐Mo_2_C/NC	52.1 µg h^−1^ mg^−1^	36.9	1.3/7.4	eN_2_RR	[99]
a1‐Ru/CNT	10.49 µg h^−1^ mg^−1^	17.48	4.7/10.9	eN_2_RR	[108]
a‐Au/CeO*_x_*–RGO	8.3 µg h^−1^ mg^−1^	10.10	7.1/NA	eN_2_RR	[161]
Bi_4_V_2_O_11_/CeO_2_	23.21 µg h^−1^ mg^−1^	10.16	2.8/NA	eN_2_RR	[162]
a1‐Ru/CNT	145.1 µg h^−1^ mg^−1^	80.62	2.1/1.9	eNO_3_RR	[108]
1T‐MoS_2_	≈20 µmol h^−1^ cm^−1^	88.12	6.7/3.2	eNO_3_RR	[163]

^a)^ NH_3_ yield rate at the optimal potential;

^b)^ FE at the optimal potential;

^c)^ Fold increase in performance after phase engineering.

**Table 5:** Comparison of EAS performance of recent electrocatalysts enhanced by coordination environment modulation (NA represent not available).

Electrocatalysts	NH_3_ yield rate^a)^	FE [%]^b)^	Increase fold (NH_3_ yield rate/FE)^c)^	Method	Refs.
Bi_1_Pd	33.8 mg h^−1^	99.6	5.6/1.5	eNO_3_RR	[111]
PR‐CuNC	130.71 mg mg_Cu_^−1^ h^−1^	94.61	3.4/1.2	eNO_3_RR	[132]
Fe−N/P−C	17 980 µg h^−1^ mg^−1^	90.3 %	2.4/1.4	eNO_3_RR	[133]
Ru_1_−TiO*_x_*/Ti	22.2 mol g^−1^ h^−1^	87.6	NA/2.4	eNO_3_RR	[134]
Rh@Cu‐0.6 %	21.61 mg h^−1^ cm^−2^	93	1.4/1.1	eNO_3_RR	[135]
Fe SAC	≈20 000 µg h^−1 ^mg^−1^	≈75	1.7/NA	eNO_3_RR	[136]
Co‐CNP	433 µg h^−1^ cm^−2^	92	1.1/1.1	eNO_3_RR	[167]
Ru‐LCN	45.02 µmol h^−1^ mg^−1^	65.96	1.8/1.8	eNORR	[137]
CuFe DS/NC	112.52 µmol cm^−2^ h^−1^	90	1.6/1.2	eNORR	[138]
Ru_0.05_Cu_0.95_	17.68 µmol cm^−2^ h^−1^	64.9	3.1/2.0	eNORR	[171]

^a)^ NH_3_ yield rate at the optimal potential;

^b)^ FE at the optimal potential;

^c)^ Fold increase in performance after coordination environment modulation.

## Conflict of Interest

The authors declare no conflict of interest.
